# Structural features of DNA that determine RNA polymerase II core promoter

**DOI:** 10.1186/s12864-016-3292-z

**Published:** 2016-11-25

**Authors:** Irina A. Il’icheva, Mingian V. Khodikov, Maria S. Poptsova, Dmitry Yu. Nechipurenko, Yury D. Nechipurenko, Sergei L. Grokhovsky

**Affiliations:** 1 Engelhardt Institute of Molecular Biology, Russian Academy of Sciences, Moscow, Russia; 2 Department of Physics, Moscow State University, Moscow, Russia

**Keywords:** Local DNA structure, RNA polymerase II promoter sequences, Sequence-specific ultrasonic cleavage

## Abstract

**Background:**

The general structure and action of all eukaryotic and archaeal RNA polymerases machinery have an astonishing similarity despite the diversity of core promoter sequences in different species. The goal of our work is to find common characteristics of DNA region that define it as a promoter for the RNA polymerase II (Pol II).

**Results:**

The profiles of a large number of physical and structural characteristics, averaged over representative sets of the Pol II minimal core promoters of the evolutionary divergent species from animals, plants and unicellular fungi were analysed. In addition to the characteristics defined at the base-pair steps, we, for the first time, use profiles of the ultrasonic cleavage and DNase I cleavage indexes, informative for internal properties of each complementary strand.

**Conclusions:**

DNA of the core promoters of metazoans and *Schizosaccharomyces pombe* has similar structural organization. Its mechanical and 3D structural characteristics have singular properties at the positions of TATA-box. The minor groove is broadened and conformational motion is decreased in that region. Special characteristics of conformational behavior are revealed in metazoans at the region, which connects the end of TATA-box and the transcription start site (TSS). The intensities of conformational motions in the complementary strands are periodically changed in opposite phases. They are noticeable, best of all, in mammals. Such conformational features are lacking in the core promoters of *S. pombe*. The profiles of *Saccharomyces cerevisiae* core promoters significantly differ: their singular region is shifted down thus pointing to the uniqueness of their structural organization. Obtained results may be useful in genetic engineering for artificial modulation of the promoter strength.

**Electronic supplementary material:**

The online version of this article (doi:10.1186/s12864-016-3292-z) contains supplementary material, which is available to authorized users.

## Background

In eukaryotes transcription initiation starts when Pol II in complex with transcription factors (TFs) interacts with the core promoter [1–3]. TATA box binding protein (TBP) subunit of the transcription factor II D (TFIID) initiates the assembly of a transcription complex. It binds to the eight base-pair TATA-element and induces local structural changes, previously characterized as a formation of the novel form of the double helix, the so-called TA-form of DNA, remarkable feature of which is extremely high base-pair inclination [4]. This leads to the sharp turn in DNA curve at the boundary of the complex. Such structural changes promote the open complex formation at the region around TSS.

X-ray structures of the complexes of TBP from *A. thaliana*, *S. cerevisiae* and *H. sapiens* with oligonucleotides that contain octanucleotides TATAAAAG or TATAAAAA in their center show that eight base-pair TATA-element binds to the concave surface of TBP by bending towards the major groove [5–8]. This causes wide opening of the shallow minor groove, which forms predominantly hydrophobic interface with the entire under-surface of the TBP saddle. The severe bend and a positive writhe radically alter the trajectory of the flanking B-form DNA, producing sharp kinks at both ends of the sequences. Fine analysis of 8 base-pair TATA box [8] has revealed remarkable structural inhomogeneity of DNA in the complex and lead to the assumption that binding polarity of TBP could be due to the asymmetry in the deformability of two halves of the recognized sequences [8–10].

Over the past decade a high variability of nucleotide sequences in the core promoters has become evident. The classic TATA-box consensus in metazoans (TATAWAAR) is found only in 10–20% core promoters [3]. Nevertheless, TBP in the complex with TFIID always binds eight base-pair DNA fragment around the positions in the range of −34– −24 bp from the TSS, depending on the species, and initiates the process of transcription.

Different methodological approaches were developed to study the complexity of the processes that occur during the formation of the transcription complex [11–17]. Analyses of various mechanical and thermodynamic properties of DNA near promoter regions were performed earlier in several works [18–21]. It was found that in the vicinity of the TSS these properties noticeably deviate from the average level.

Evolution of core promoters had to select nucleotide sequences with structural properties important for TBP recognition and subsequent formation of the open transcription complex in such a way as to provide an optimal level of transcription. The goal of our work is to unravel the reason why eukaryotic transcription machineries recognize a nucleotide sequence as the core promoter, despite the differences in their textual motifs, in particular, despite the absence of TATA-box consensus in most promoters. What structural and conformational properties, inherent to the naked DNA, are significant in this process? The answers to these questions may be beneficial to the different application goals of molecular biology.

We have found that common regularities of the core promoter architecture in each species may be revealed after superposition of signals from a huge amount of species’ promoter sequences properly aligned at TSS. Profiles of the signals of physical, mechanical and 3D structural characteristics show alterations of these properties along the nucleotide sequences in each species. In our analysis we for the first time in addition to the well-known DNA characteristics, which numerical parameterization at the level of ten different base-pair steps are collected at the database DiProDB http://diprodb.fli-leibniz.de [22], have used the indexes that reflect the intensities of sequence-specific ultrasonic cleavage at the di- and tetranucleotide levels [23, 24] and the new data on the propensities of hexanucleotides to the DNase I cleavage [25]. These characteristics provide information on the internal properties of each complementary strand, in other words they allow register autonomous behaviour of each strand in double-stranded DNA. The intensity of ultrasonic cleavage is dependent on the intensity of conformational movement of the sugar-phosphate backbone [23] so it provides information on the dynamic states of the sequences.

## Results and discussion

The sets of nucleotide sequences from the core promoter regions of six metazoans and two unicellular eukaryotic species, aligned at the TSS, were retrieved from the EPD New section of the Eukaryotic Promoter Database (EPD) (http://epd.vital-it.ch) [26]. EPD New is a collection of experimentally validated promoter sequences for selected model organisms. Their TSS-mapping was the result of high-throughput experiments such as CAGE and oligo-capping. We have used sets of the animal promoters (23,360 promoters for *H. sapiens,* 21,239 promoters for *M. musculus*, 15,073 promoters for *D. melanogaster*, 10,726 promoters for *D. rerio* (some sequences were discarded as their length was shorter than 60 nucleotides), 7120 promoters for *C. elegans*; plant promoters (10,229 promoters for *A. thaliana*) and fungi promoters (4324 promoters for *S. cerevisae* and 3440 promoters for *S. pombe*). We have checked that all these sequences are 60 nucleotides long and strictly defined.

### Comparative statistical characteristics of the nucleotide sequences in the core promoters of metazoans and unicellular fungi *S. cerevisiae* and *S. pombe*

**Fig. 1:**
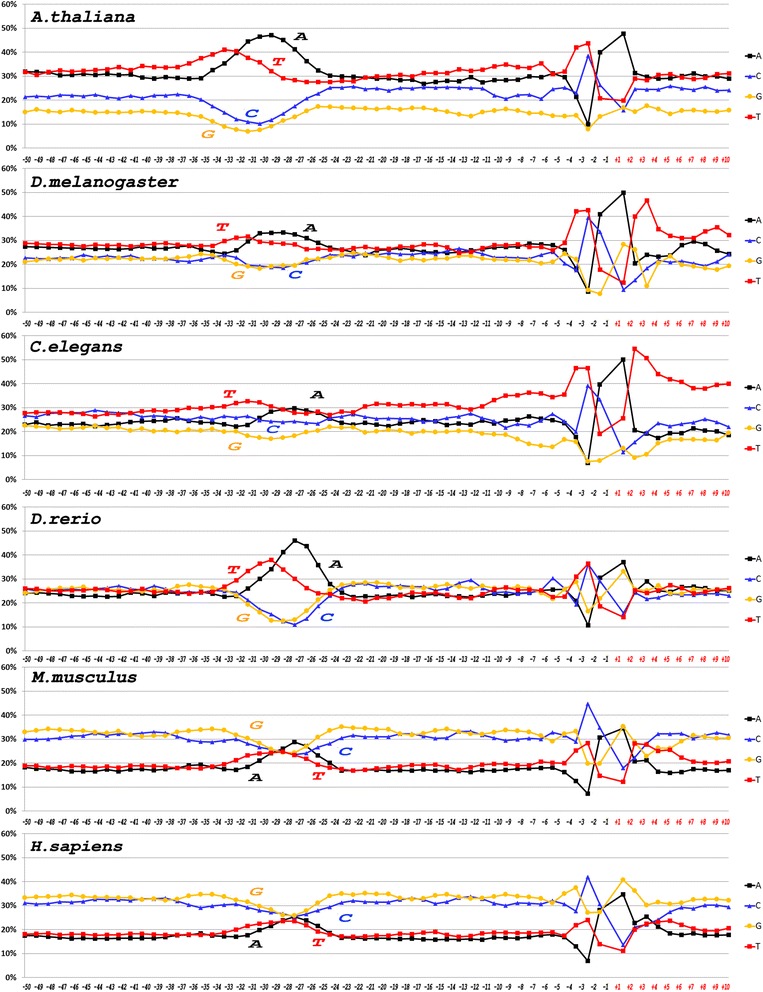
Profiles of core promoter sequences as the mononucleotides frequencies of occurrence (in percentages) at each position along the strand, complementary to template for data sets of *A. thaliana, D. melanogaster, C. elegans, D. rerio*, *M. musculus* and *H. sapiens*

**Fig. 2:**
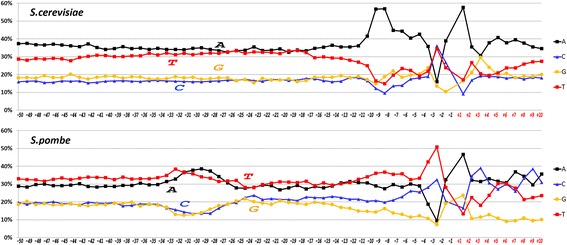
Profiles of core promoter sequences as the mononucleotides frequencies of occurrence (in percentages) at each position along the strand, complementary to template for data sets of *S. cerevisiae* and *S. pombe*

The frequencies of mononucleotide occurrences at each position along the strand, complementary to the template (namely the strand with 5′→3′ vector directed to the TSS from the upstream region; hereinafter we will refer to it as the upper strand) for six metazoan species are shown in Fig. 1 and for unicellular fungi *S. cerevisiae* and *S. pombe* in Fig. 2. Logo-representation of the sequences [27] is shown in the Additional files 1 and 2, respectively.

The differences between nucleotide sequences of metazoan species at the mononucleotide level (Fig. 1) are attributed mainly to the relative content of A and T (**W** nucleotides in IUPAC nomenclature), and G and C (**S** nucleotides in IUPAC nomenclature). For mammal promoters (*M. musculus, H. sapiens*) the percentage of **S** exceeds that of **W** in all positions, for the exception of the positions −30 to −26 bp, where the percentages of **W** are almost equal to that of **S**. On the opposite, *A. thaliana* promoter sequences have the highest percentage of **W** nucleotides at all positions with the maximum in the region from −35 to −26 bp. Promoter sequences of three other species, *D. melanogaster*, *C. elegans* and *D. rerio* are composed from roughly equal amount of **W** and **S** nucleotides, while the region from −31 to −26 bp is also enriched by **W** nucleotides.

The mean content of **W** nucleotides in the core promoters of both unicellular fungi and in *A. thaliana* is appreciably higher than that of **S** nucleotides (Figs. 1 and 2). Noteworthy, the distribution of purines and pyrimidines between the complementary strands in two fungi species is different. While in *S. cerevisiae* the upper strand is enriched by A (thereby the template strand is enriched by T), in *S. pombe* the upper strand is enriched by T. Moreover, composition of **S** nucleotides in the upper strand of *S. pombe* also is more frequently represented by C than by G. Thereby the template strand of *S. pombe* is enriched by A and G.

**Fig. 3:**
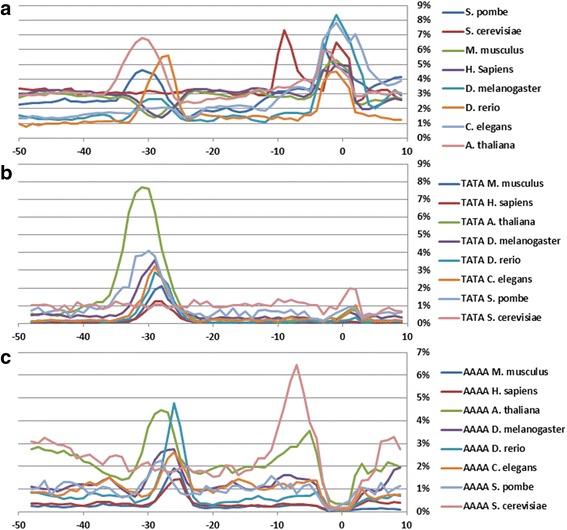
**a** Standard deviations of dinucleotide distributions at each position of core promoter sequences of *A. thaliana, D. melanogaster, C. elegans, D. rerio*, *M. musculus, H. sapiens, S. cerevisiae* and *S. pombe*. **b** Frequencies of occurrence of tetranucleotide TATA in core promoter sequences of *A. thaliana, D. melanogaster, C. elegans, D. rerio*, *M. musculus, H. sapiens, S. cerevisiae* and *S. pombe*. **c** Frequencies of occurrence of tetranucleotide AAAA in core promoter sequences of *A. thaliana, D. melanogaster, C. elegans, D. rerio*, *M. musculus, H. sapiens, S. cerevisiae* and *S. pombe*

Distributions of dinucleotides in the core promoter sequences of all eight species are shown in Additional file 3 (a–f). The profiles of all species except for *S. cerevisiae* have two regions where frequencies of dinucleotide occurrences deviate from the mean values. These two regions are located at the TATA-box position and at the region around TSS. Profiles of the standard deviations of the dinucleotide distributions in the core promoter sequences of all species are presented in Fig. 3a. These profiles characterize the abmodality of dinucleotide distribution in different parts of core promoters in different species. A low standard deviation indicates that the data points tend to be close to the expected value of the set while a high standard deviation indicates that the data points are spread out over a broader range of values. In mammal promoters the TATA-box position corresponds to the minimum of the standard deviation of the dinucleotide distribution while in other metazoans it corresponds to the maximum. This could be the consequence of two factors: a) the relative content of the **W** and **S** nucleotides in the promoters of the species and b) the irregularity of the distribution of dinucleotides in the promoter sequences. The profile of *S. pombe* resembles the profile of *A. thaliana*; this cannot be said about the profile of *S. cerevisiae*. Its singular region for the dinucleotide distribution is shifted downstream to the TSS. These differences in core promoters of two yeasts may be associated with an evolutionary distance between *S. pombe* and *S. cerevisiae. S. pombe* diverged from *S. cerevisiae* 500 million years ago [28].

Frequencies of tetranucleotide occurrences in terms of “Py,Pu” (Additional file 4 (a–e) and Additional file 5: Archive S1 of excel-spreadsheets) show the pronounced species-specific differences. They are observable at all positions but are the greatest in the regions of the TATA-box and the TSS.

The distributions of TATA and AAAA are of special interest. In the core promoters of mammals, *D. melanogaster*, *D. rerio* and *C. elegans* the occurrence of TATA reaches its maximum at the positions −28 or −29 bp, while in *A. thaliana* it is shifted to the position −31 bp relative to the TSS (Fig. 3b). The maximum of the occurrence of AAAA falls on the position −26 bp in mammals, *D. melanogaster*, *D. rerio* and *C. elegans*, while in *A. thaliana* it falls on −28 bp (Fig. 3c). Profound differences are observed between TATA and AAAA distributions in the promoters of two fungi. While the peaks of occurrence for TATA and AAAA in *S. pombe* follow each other (they correspond to −30 and −29 bp, respectively), in *S. cerevisiae* both peaks are disposed in the reversed order near TSS (AAAA at −7 bp, and TATA at +1 bp). These findings may only partially help to locate the position of TBP binding. Genome-wide analysis of *A. thaliana* promoters with a remarkably high AT-content show that only 29% of promoters contain TATA motif clustered around the position −32 bp with respect to the TSS [29].

Distributions of dinucleotides and tetranucleotides in the region around TSS possess a rather high degree of species-specific diversity (Additional files 3 (a–f), 4 (a–e)). Logo-representation also points to the variability of Inr consensus sequences (Additional files 1 and 2) while PyPu step in the positions −1, +1 is undoubtedly functionally significant. The +1 position is mainly occupied by A in all species except for *M. musculus* (A in 7347 promoters, G in 7510 promoters) and *H. sapiens* (A in 8088 promoters, G in 9519 promoters). The DeepCAGE genome-wide analysis of core promoter structure for S*. pombe* [30] enabled identification of over 8000 core promoters and identified Inr consensus sequence PyPyPuN(A/C)(C/A). Logo-representation for the set of 3440 promoters of *S. pombe* is in agreement with this consensus (Additional file 2). The only discrepancy is at the position −2 bp where occurrence of Py is somewhat higher than that of Pu.

### Local variations of physical and structural properties of the naked DNA in the core promoter region

The numerical indexes of local physical and 3D structural characteristics for ten base-pair steps in DNA were chosen from the dinucleotide property database (http://diprodb.fli-leibniz.de). It contains several versions of parameters of the same name and we verified that the profiles built from different versions of parameters are in qualitative agreement with each other. Here we present the profiles that were constructed using the latest versions of the parameters.

**Fig. 4:**
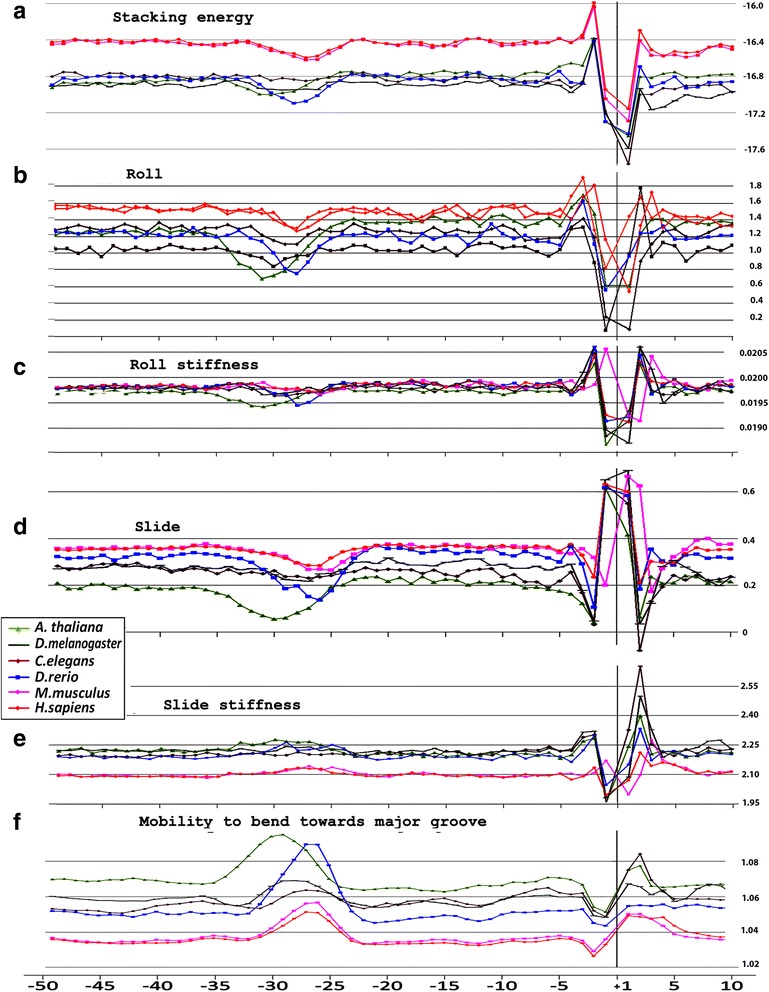
Local variations of the values of physical and structural parameters in core promoter regions of *A. thaliana, D. melanogaster, C. elegans, D. rerio*, *M. musculus* and *H. sapiens.* **a** Stacking energy (in kcal/mol). **b** Roll (in degrees). **c** Stiffness of the duplex structure to Roll alteration (in kcal/mol degree). **d** Slide (in angstroms). **e** Stiffness of the duplex structure to Slide alteration (in kcal/mol angstrom). **f** Mobility to bend towards major groove (in mobility units)

**Fig. 5:**
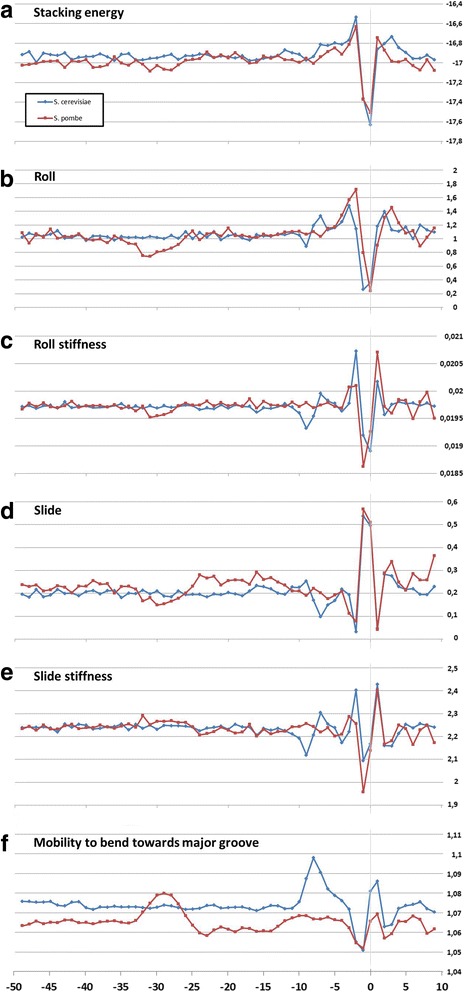
Local variations of the values of physical and structural parameters in core promoter regions of *S. cerevisiae* and *S. pombe.* **a** Stacking energy (in kcal/mol). **b** Roll (in degrees). **c** Stiffness of the duplex structure to Roll alteration (in kcal/mol degree). **d** Slide (in angstroms). **e** Stiffness of the duplex structure to Slide alteration (in kcal/mol angstrom). **f** Mobility to bend towards major groove (in mobility units)

We analyzed profiles of six physical and structural parameters, which characterize DNA duplex as a whole – the stacking energy, the base-pair step parameters Roll and Slide, the stiffness’s of the structure to Roll alteration and to Slide alteration, as well as their stiffness to bend towards major groove that includes alteration of all base-pair steps parameters. The corresponding profiles are presented in Fig. 4(a–f) for metazoans, and in Fig. 5(a–f) for unicellular fungi. Profiles of variations of stacking energy (Figs. 4a and 5a) and the base-pair step parameters Roll and Slide (Figs. 4b, d and 5b, d) are presented in the parametrization of Perez et al. [31], the profiles of stiffness’s variation in the DNA double helix to Roll and Slide changes (Figs. 4c, e and 5c, e) are presented in the parametrization of Goni et al. [32]. These five parameters describe DNA at the base-pair step resolution.

***Stacking energy*** is a part of an enthalpy of DNA formation and defines its stabilizing forces. The stacking energy profiles for *M. musculus* and *H. sapiens* are quite identical in the core promoter regions while the profiles of four other metazoan species, *A. thaliana, D. melanogaster, C. elegans, D. rerio* (Fig. 4a)*,* and both fungi (Fig. 5a) are shifted downwards. The shallow global minimum of base stacking energy around the position −30 bp relative to the TSS is present at the profiles of all species except for *S. cerevisiae* (Figs. 4a and 5a). It indicates that base-pair stacking is preserved in the naked DNA in the TATA-box region. At the same time the melting temperature as well as the entropy reaches the global minimum in these positions. The entropy minimum points to the high extent of ordering in the TATA-box region of the naked DNA (the profiles of entropy built in parameterization of two different groups of authors [33, 34] for *H. sapiens* core promoters are presented in Additional file 6).

***Base-pair step parameter Roll*** defines an angle between the average planes of two neighboring base-pairs. The positive value of this angle corresponds to its opening towards the minor groove. Among the three rotational parameters (helical Twist, Roll and Tilt) the Roll is the most important for understanding the bending of DNA [10]. Our profiles show that in all the species the Roll parameter in the core promoter is positive but rather small (0.8–1.6°) and at the TATA-box position its value is slightly reduced in all species except for *S. cerevisiae* (Figs. 4b and 5b) while the stiffness of the duplex to Roll variation relaxes (Figs. 4c and 5c). This means that if local Roll alterations are necessary for TBP binding they are not energetically expensive.

***Base-pair step parameter Slide*** defines the mutual displacement of the neighboring base pairs in the direction perpendicular to the minor and major grooves. Positive Slide values are a distinguishing feature of B-DNA while in the A-form of DNA the values of the Slide are always negative. Thus, the sign of the Slide is an important indicator, that allow to discriminate B- and A-DNA forms [35]. Our profiles show that in all the species the Slide values are always positive but rather small and became somewhat smaller in all the species except for *S. cerevisiae* at the position of the TATA-box (Figs. 4d and 5d) while the stiffness of the duplex to Slide variation, unlike the stiffness to Roll variation, at these positions is slightly increased (Figs. 4e and 5e). This means that if local Slide alterations are necessary for TBP binding, they are energetically expensive as opposed to the Roll. Possible functional role of DNA transformation from B-form to TA-form [4] during complex formation lead us to the conclusion that an equilibrium value of the Slide for the binding of TBP may have an influence on the rate of the complex formation. As B-form DNA is always characterized by the positive value of the Slide, only high positive Roll may increase the likelihood of transformation to the structure with the negative values of the Slide [36, 37]. So we propose that the sequence selection for the region that binds to TBP has to be directed to the lowering duplex stiffness to Roll variations. Conformational properties of the sequences are of great importance in the natural selection. A striking example is the significant decrease of the frequency of occurrences of dinucleotides GG and CC in comparison to the other CG-containing dinucleotides in the position of TATA-box of all species (Additional file 3 (a f)). The exception is *S. cerevisiae*. It is the consequence of clustering of the distributions of Roll and Slide values in GG/CC step in DNA duplex [36] where one of the clusters refers to A-family forms of DNA and the other to B-family. The energetically unfavorable region between two clusters impedes the reversibility of the transformation from B-family to A-family forms (TA-form belongs to A-family). So GG/CC step unfit for promoter functioning. In contrast, the distributions of Roll and Slide values in the duplex structure in AA/TT step do not reveal such clustering [36] so this step can well contribute to the reversibility of the B↔TA conformational changes.

All profiles lose their smoothness around TSS so despite the nucleotide diversity in this region (see Additional files 1, 2, 3 and 4(a-e)) their structural characteristics at the base-pair step level are very similar. We can only speculate that selection of these sequences was directed to obtain the most irregular duplex structures that can facilitate unwinding around the TSS.

***Mobility to bend towards major groove*** in the parametrization of Gartenberg and Crothers [38] was resolved for all 16 dinucleotides and relates to each of the complementary strands. At the Figs. 4f and 5f this characteristic is presented for the upper strand (the strand complementary to the template). In all species, except for *S. cerevisiae,* the profiles of the structural mobility are similar: its value noticeably increased around the TATA-box position. At the profile of *S. cerevisiae* similar increase of mobility to bend towards major groove falls in to the region around the position −8 bp relative to the TSS.

### Detailed comparison of the profiles of unicellular fungi S. cerevisiae and S. pombe

Figure 5 (a–f) shows that alteration of all properties along the promoter sequence is significantly different between two variants of fungi. All *S. pombe* profiles preserve positions of extremum typical for metazoans (that is in the region from −30 to −25 bp relative to TSS), and step by step variations at the positions from −3 to +3 bp around TSS that correspond to the position of Initiator element (Inr). These data are in accordance with the results of DeepCage analysis [30] that revealed similarity of organization of *S. pombe* core promoters with that of higher eukaryotes. In contrast to *S. pombe*, at the profiles of *S. cerevisiae* all the properties only slightly oscillate around their average values prior to the position −11 bp and none of the properties has an extremum falling in the region from −30 to −25 bp. For *S. cerevisiae* extremum positions for all characteristics appear only nearby the TSS, namely at the position −8 bp. Thus, in addition to the divergence of nucleotide sequences we have described above, the architecture of core promoters of these two yeast species is also different.

### Local variations of ultrasonic cleavage and DNase I cleavage intensities in promoter sequences

***Profiles of ultrasonic cleavage indexes*** provide information about the intensity of sugar-phosphate dynamics along the core promoter sequences in each of the complementary strands.

The permanent atomic movement around single bonds in DNA may be represented as coupling of S↔N interconversion in a sugar ring [39–42], epsilon/dzeta changes (BI↔BII motion) [43–46], alfa/gamma flips [47, 48] and mutual rotation of sugar around the glycoside bond [49]. NMR data show that positions of N↔S equilibrium and BI↔BII equilibrium are interdependent and that the dynamics of conformational changes occur on the picosecond to nanosecond timescale [50]. Populations of N- and S-forms are dependent on the type of nucleoside. The minor population of the N-forms is higher for pyrimidines (at a greater extent in cytidine) than for purines [51]. It was shown that repuckering in cytidine furanose ring with a time constant of around 100 ps is the most rapid conformational motion in the sugar-phosphate backbone [52]. These data when compared with the ultrasonic cleavage rates lead us to the conclusion: the higher is the rate of the sugar ring interconversion on the 5′-end of a dinucleotide, the more intensive is the cleavage. Contextual dependence is a consequence of the sequence-specific conformational exchange between N↔S equilibrium and BI↔BII equilibrium. For description of the intensity of ultrasonic cleavage at different positions we use several indexes, namely ***R*** – relative cleavage intensities of central position of each of 16 dinucleotides; ***T*** –relative cleavage intensities of central position of each of 256 tetranucleotides; ***S*** – the combination R and T, namely the difference between the tetranucleotide cleavage index and the dinucleotide cleavage index in its center (***S*** = ***T*** – ***R***). If S < 0 the first and the fourth nucleotides of a tetranucleotide brings down the intensity of the cleavage in the central step, otherwise they increase it. Relative cleavage intensities of each of 16 dinucleotides (***R*** index) and relative cleavage intensities of each of 256 tetranucleotides (***T*** index) were obtained from experiments of DNA fragmentation by ultrasound [23]. The products of ultrasound irradiation of plasmid DNA restriction fragments with known sequences were separated by gel electrophoresis technique with subsequent computer digitization of the gel band densities and statistical treatment of more than 20 500 relative cleavage intensities [23, 53, 54]. One can see that the intensities of the cleavage in complementary di- or tetranucleotides are different if their base-paired fragment is asymmetrical. For example, ***R*** index for CA is 1.130, while for TG it is 0.900; ***T*** index for ACGA is 1.537, while for TCGT it is 1.263.

It should be emphasized that the observed sequence specificity of ultrasonic cleavage is typical only for the double-stranded B-DNA structure that imposes definite conformational regularities as it was mentioned above. Single-stranded DNA nick leads to the loss of these conformational regularities and changes the character of ultrasonic cleavage [55]. Later we analyzed genomic reads from the NGS data and discovered similar sequence specificity of DNA fragmentation produced by the methods, based on the action of hydrodynamic forces [56].

**Fig. 6:**
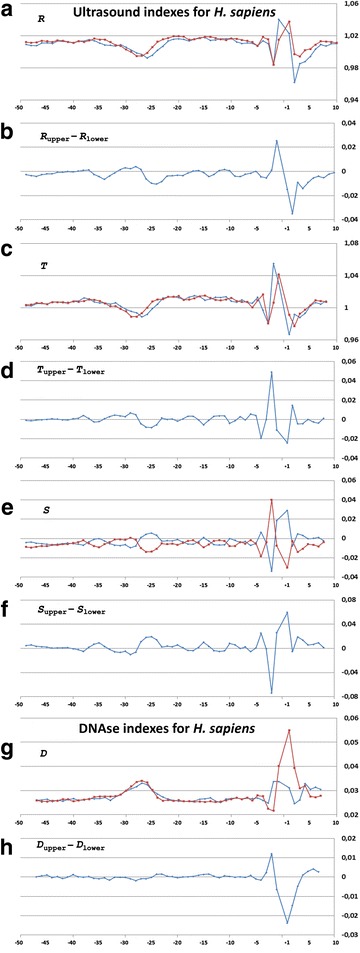
**a**–**h** Profiles of ultrasonic cleavage indexes and DNase I cleavage indexes for *H. sapiens* core promoters

**Fig. 7:**
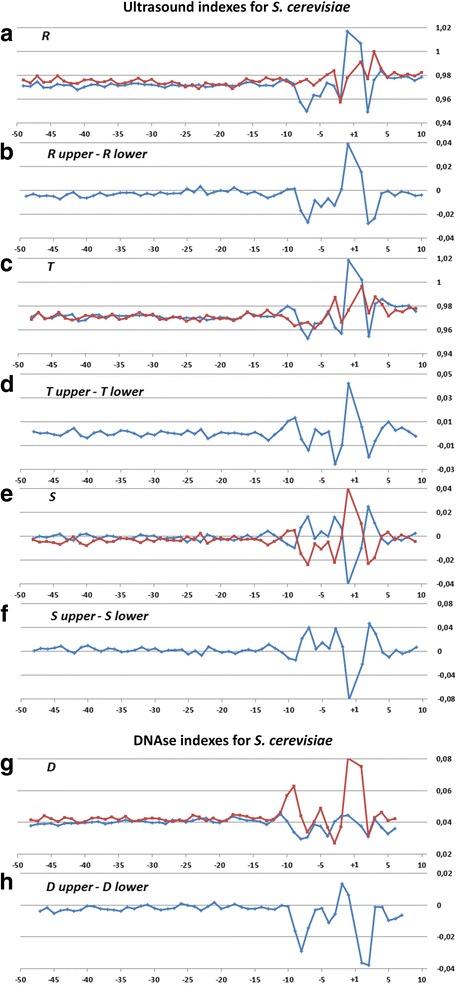
**a**–**h** Profiles of ultrasonic cleavage indexes and DNase I cleavage indexes for *S. cerevisiae* core promoters

**Fig. 8:**
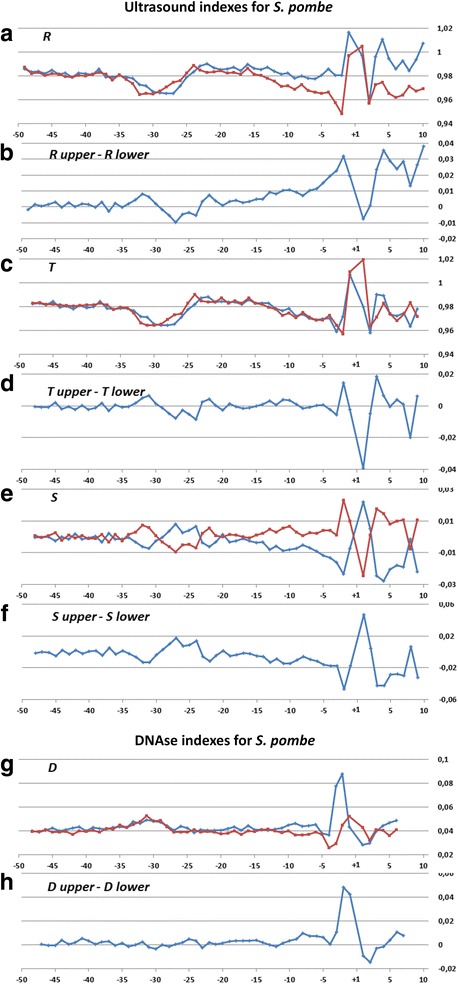
**a**–**h** Profiles of ultrasonic cleavage indexes and DNase I cleavage indexes for *S. pombe* core promoters

**Fig. 9:**
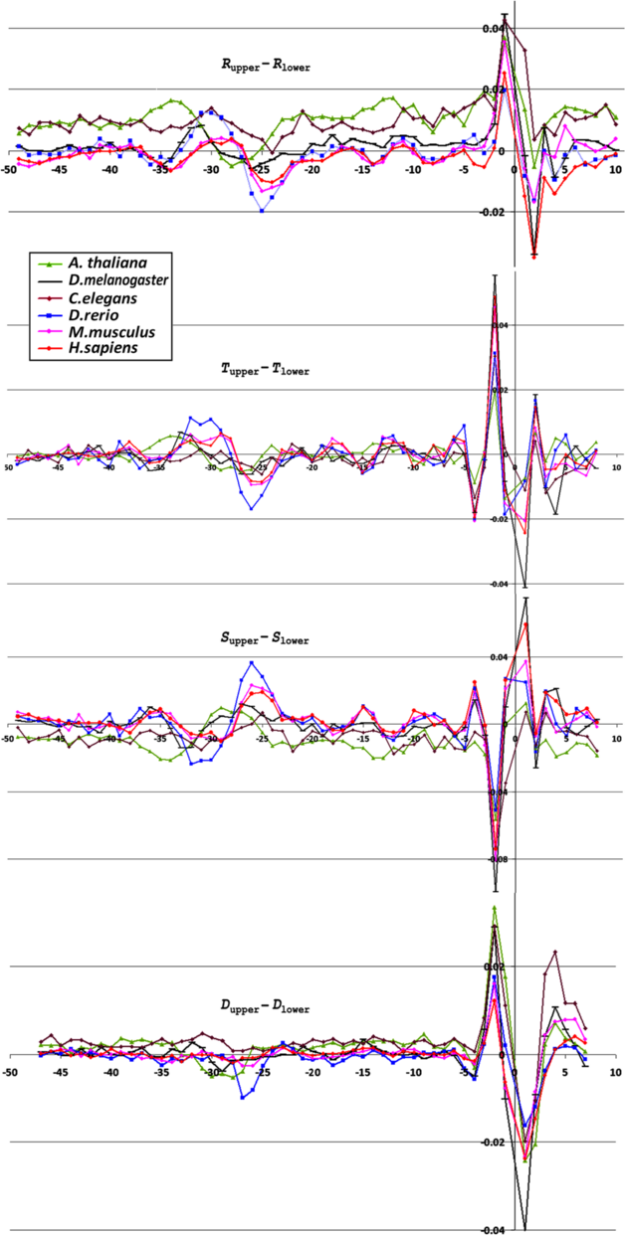
Differences between complementary strands profiles for ultrasonic cleavage indexes and DNase I cleavage indexes in core promoters *A. thaliana*, *D. melanogaster*, *C. elegans*, *D. rerio, M. musculus* and *H. sapiens*

Profiles of the ultrasonic cleavage indexes for *H. sapiens*, *S. cerevisiae* and *S. pombe* core promoters are presented in Figs. 6, 7 and 8 *,* respectively. The ultrasonic cleavage profiles for both complementary strands in the core promoters of *A. thaliana, D. melanogaster, C. elegans, D. rerio* and *M. musculus* are shown in Additional files 7, 8, 9, 10 and 11 while differences between the complementary strands for these species are presented in Fig. 9. The profiles of the indexes ***R*** *,* ***T*** and ***S*** for the upper strand are depicted in blue and for the lower (template) strand in red.

The lowest value of the cleavage rates for *H. sapiens* core promoters is detected in the region from −32 to −24 bp relative to TSS. This reflects a decrease of conformational motion in this region. The differences in the cleavage rates between complementary strands, shown for *H. sapiens* in Fig. 6b and d, change the sign with the periodicity of about 5 bp between the positions −23 and −3 bp relative to the TSS. All profiles lose their smoothness around TSS. It may be consequence of the characteristic properties of Inr consensus. We have proposed above that irregularity of this region may facilitate local unwinding. The ***S***-index that reproduces the influence of the nearest context on the intensity of cleavage is shown in Fig. 6e. In the region between positions from −27 to −22 bp relative to TSS the cleavage rate of the lower strand is decreased indicating a decrease of the conformational motion in that region. At the same time the cleavage rate of the upper strand is increased indicating an increase of the conformational motion. The profiles of the differences of the ***S***-indexes revealed periodic alteration of the conformational motion intensity in the complementary strands until the position of −3 bp (Fig. 6f).

Positions of the minima for the ultrasonic cleavage in di- and tetra-resolution at *S. pombe* profiles, presented in Fig. 6(a,c), are similar to the positions of the minima in metazoans but they are shifted by two steps upstream as compared to *H. sapiens*. In *S. pombe* the decrease of sugar-phosphate conformational dynamics – one of the distinguishing features of the TATA-box – emerges at the region of −34– −26 bp relative to the TSS. An important distinction between the ultrasonic cleavage profiles of *S. pombe* and metazoans is an absence of periodicity of the differences of the cleavage intensities of the complementary strands that we observed in the profiles of metazoans between the TATA-box and the position −3 bp relative to TSS. This distinguishes profiles of *S. pombe* from the profiles of metazoans. The profiles of ***T***-index in both complementary strands of *S. pombe* shown in Fig. 8c are placed practically at the same level. This is the result of a high degree conformational homogeneity of *S. pombe* core promoter sequences. Moreover, variations of the ***S***-index (Fig. 8e) show that overall level of the conformational motion intensity in the complementary strands is preserved due to the proper choice of the adjacent nucleotides. We compared this finding with the results of the study of biochemical and genetic system of *S. pombe* [23], which showed that in vitro initiation of the transcription in *S. pombe* is directed by the unique scanning mechanism, preferentially using purines, within a narrow window approximately 25–40 bp downstream from the edge of the TATA element. The peculiarity of the structure of the TFIIB in *S. pombe* defines the specific character of their core promoter sequences: the template strand possesses a high level of the purine nucleotides. The frequencies of the purine nucleotides in the template strand of *S. pombe* are significantly higher than that of *S. cerevisiae* (Fig. 2; Additional file 2). So we can speculate that registered periodicity of the differences in conformational motion intensity in complementary strands starting from the edge of the TATA-box may determine some functionality for transcription initiation in metazoan.

***Profiles of DNA cleavage by DNase I*** indicate the minor groove width variation because this structural characteristic of DNA has the greatest influence on the cleavage by DNase I [25]. Figures 6g, 7g and 8g show the profiles of the DNase I cleavage in hexanucleotide resolution for the upper (blue) and the lower (red) strands for *H. sapiens, S. cerevisiae* and *S. pombe* core promoters, accordingly, while the differences between the cleavage indexes of the complementary strands are shown in Figs. 6h, 7h and 8h. Widening of the minor groove in TATA-box positions in the *H. sapiens* and *S. pombe* core promoters are evident. But the differences between the DNase I cleavage rates in the complementary strands for *H. sapiens* are less pronounced in comparison with the ultrasonic cleavage. They are significant only near the TSS starting from the −5 bp position. The DNase I cleavage profiles for both complementary strands in the core promoters of *A. thaliana, D. melanogaster, C. elegans, D. rerio* and *M. musculus* are shown in Additional files 7, 8, 9, 10 and 11; the differences between the complementary strands for these species are presented in Fig. 9.

The shapes of the ultrasonic and DNase I cleavage profiles of *S. cerevisiae* core promoters (see Fig. 7(a-h)) are noticeably different from the profiles of all metazoans and *S. pombe*. None of the indexes deviates from the average level in the region from −50 up to −15 bp relative to the TSS. Taking into account the lack of singularities at the region near −30– −25 bp relative to the TSS in all studied profiles of *S. cerevisiae* core promoters, we conclude that its TBP-binding position is located elsewhere. The characteristic shape of the profiles around the position −8 bp (the positions of the extremums for all properties) points to the high probability of interaction between DNA and regulatory molecules at the vicinity of the TSS in *S. cerevisiae* core promoters.

Periodical (in antiphase) variation of the ultrasonic cleavage intensities in the complementary strands is well discernible at the profiles of *H. sapience*, *M. musculus* and *D. rerio* but to a lesser extent for all other species. Both methods generate quite identical profiles for *M. musculus* (Additional file 11) and *H. sapiens* (Fig. 6) while the profiles of other species have their own specificity: the maximum and minimum positions are shifted upstream at the profiles of *A. thaliana* (Additional file 7) compared to the profiles of *M. musculus* and *H. sapiens*; the amplitude of oscillations of the cleavage differences is bigger for *D. rerio* (Additional file 10) and smaller for *D. melanogaster* (Additional file 8). The profiles of the DNase I cleavage rates show periodic changes of the minor groove width. Its maximum is around the positions −30– −25 bp relative to the TSS (depending on the species). Namely, in these positions the sugar-phosphate dynamics intensity reaches its minimum, which is clearly visible at the profiles of the ultrasound cleavage indexes.

So the decrease of the sugar-phosphate conformational dynamics and widening of the minor groove are distinguishing features of the TATA-box in all metazoans and *S. pombe*. The periodic variations of the conformational motion intensity starting from the edge of TATA-box up to the position of −3 bp, the amplitude of which depends on the species, are observable only in the metazoan core promoters.

### Separate consideration of TATA-containing and TATA-less promoter sequences

**Fig. 10:**
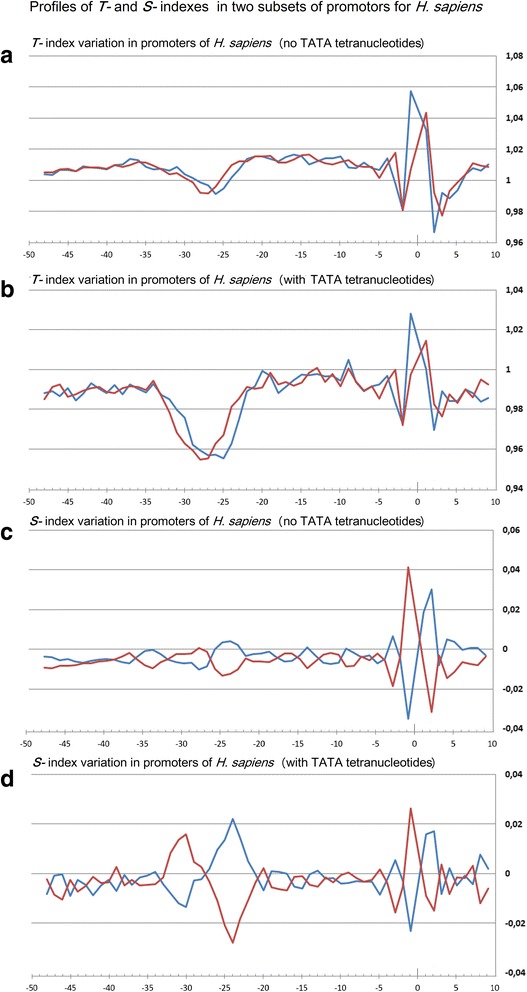
**a**-**d** Profiles of ***T-*** and ***S-***indexes in two subsets of promotors of *H. sapiens*

**Fig. 11:**
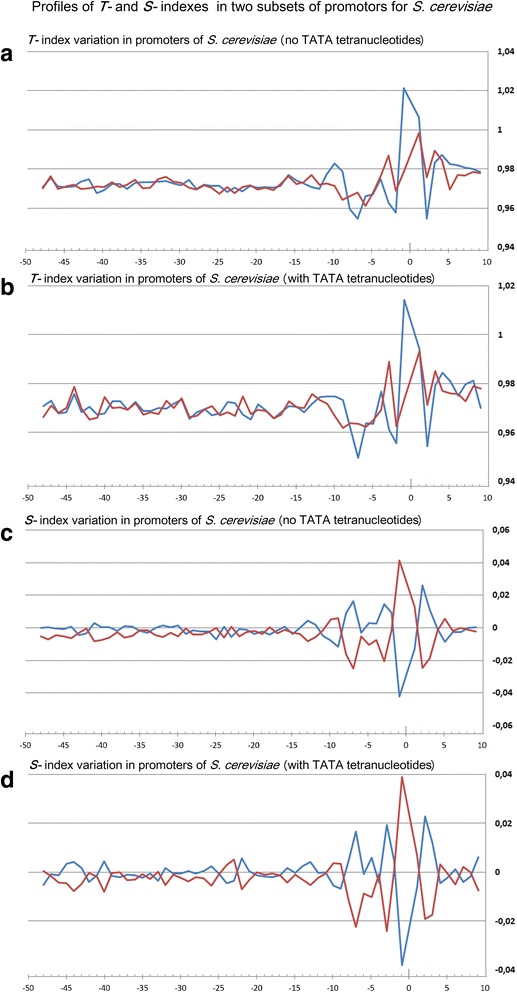
**a**-**d** Profiles of ***T-*** and ***S-***indexes in two subsets of promotors of *S. cerevisiae*

**Fig. 12:**
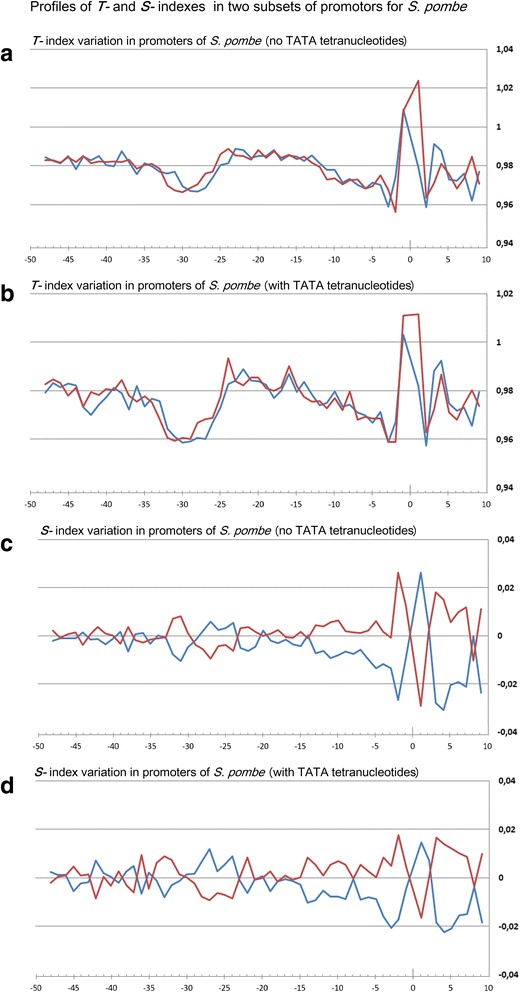
**a**-**d** Profiles of ***T-*** and ***S-***indexes in two subsets of promotors of *S. pombe*

We have analyzed possible influence of the presence of TATA tetranucleotide in promoter sequences. For this purpose we have divided promoter sets of all species into two groups. First group contain TATA tetranucleotides in any position while the second does not contain TATA tetranucleotides. The number of promoters in these two groups is listed in the Table 1. In all examined species the number of promoters without TATA exceeds the number of promoters with TATA. The set of promoters of *H. sapience* contain the smallest subset with TATA (only about 8%) while the biggest subset with TATA belongs to *S.pombe* (about 64%). It seems that the higher is species organization, the bigger is the percentage of TATA-less promoters. It should be emphasized that generally accepted category of TATA-less promoters imply the lack of consensus sequence TATAWAAR while tetranucleotide TATA may be presented at other positions, therefore the real amount of TATA-less promoters in the species is bigger. Comparison of the values of standard deviation in dinucleotide distributions (Fig. 3a) with the percentage of TATA-less promoters in the species reveal an interesting regularity. The bigger is the subset of TATA-less promoters the lower is the standard deviation of dinucleotide distribution in TATA-box region in the species. Profiles of ***T-*** and ***S***-indexes for both strands are constructed for both promoter subsets of *H. sapiens* (Fig. 10), *S. cerevisae* (Fig. 11) and *S. pombe* (Fig. 12). They show that the absence of TATA tetranucleotide does not alter the shape of the profiles and the position of the minimum for ***T-***index is preserved at TATA-box position. Profiles of ***S-***index for *H. sapiens* in addition show that the ***S-***index characteristic variations, which evidenced for periodic changes of conformational motion intensity in complementary strands, appear more pronounced for TATA-less subset. Numerical values of ***T-*** and ***S***-indexes of TATA-containing and TATA-less subsets of promoters of all examined species are provided in Additional file 12: Archive S2.

**Table 1:** The proportion of promotors with and without TATA in any positions in the whole sets of the species

	Subset with TATA (number and percentage)	Subset without TATA (number and percentage)	The set
*H. sapiens*	1859 (7.96%)	21501 (92.04%)	23360
*M. musculus*	2020 (9.51%)	19219 (90.49%)	21239
*D.melanogaster*	3983 (26.42%)	11090 (73.58%)	15073
*D. rerio*	1640 (15.29%)	9086 (84.71%)	10726
*C. elegans*	1400 (19.42%)	5720 (80.58%)	7120
*A. thaliana*	4117 (40.25%)	6112 (59.75%)	10229
*S. cerevisae*	1589 (36.68%)	2735 (63.32%)	4324
*S. pombe*	1230 (35.76%)	2210 (64.24%)	3440

## Conclusions

The present study was aimed to elucidate the special structural organization of the naked DNA in minimal core promoters of RNA polymerase II, which could be important for their functioning. We have found that a large number of physical, mechanical and 3D structural properties, which can be characterized by the numerical base-pair step indexes, give similar profiles for the metazoan and *S. pombe* core promoters despite the variations of nucleotide sequences. Singular properties of the DNA structure are observed near the position of −30 bp (with the extremums at −28, or −32 bp, depending on the species) relative to the TSS, as well as at the region around the TSS. Profiles of the ultrasonic cleavage indexes of metazoan and *S. pombe* show the decrease of intensity of ultrasonic cleavage around the position −30 bp, which reflects a significant diminution of the conformational movement of the sugar-phosphate backbone in TATA-box region while the profiles of the indexes of DNase I cleavage points to the significant widening of the minor groove in this position. At the distance of about 30 bp apart from TATA-box all physical and structural characteristics of the adjacent base-pair steps reveal high extent of irregularity at the region of about 6–8 bp.

So we can say that a naked DNA in the area of core promoters reveals the properties, which are the necessary prerequisites for the structural perturbations that facilitate TBP binding and subsequent open complex formation. The minor groove of TATA-box is expanded; DNA mobility to bend towards major groove is enhanced while stiffness to the mutual displacement of the neighboring base pairs in the direction, perpendicular to the grooves is reduced. These structural properties are found in promoter regions of all but one examined species despite the significant variations of the nucleotide sequences.

Ultrasonic cleavage profiles for metazoans show a characteristic periodicity of the intensity of the ultrasonic cleavage. It occurs in the opposite phases in the complementary strands, starting from the end of the TATA-box to the position −3 bp relative to TSS with five-step periodicity. Such periodicity of the cleavage reflects the alteration of the conformational dynamics intensity in the complementary strands between TATA-box and TSS. The degree of its amplitude depends on the species and is best noticeable in mammals. We assume that the regular alternation of areas with high and low extent of conformational movement can carry functional load. We have not observed such periodicity at the profiles of *S. pombe* core promoters; instead we observed a high level of asymmetry for the Pu/Py content in the complementary strands in that region.

Core promoters of *S. cerevisiae* have unique properties among all eukaryotic organisms studied. The singular regions in the numerical profiles, constructed for all characteristics, are shifted downstream to the position −8 bp relative to the TSS.

Thus, we have found three different patterns of Pol II core promoter architecture. The first is typical for all metazoans: the structure combines two segments with different 3D and mechanical properties, namely the region of TATA-box and TSS. They are separated from each other by two turns of the B-form helix with the unique level of conformational motion asymmetry in the complementary strands. The second variant presents the structure of the *S. pombe* core promoters, which differs from the first only at the conformational level of organization: there is no asymmetry in the intensity of the conformational motion between the complementary strands in the region between the TATA-box and the TSS. The profiles of the core promoters of *S. cerevisiae* revealed the third variant of the structural organization, which is significantly different. The singular positions are shifted down by 20 bp and fall in the region around −8 bp relative to the TSS. This fact points to the uniqueness of the structural organization of DNA in the core promoter of *S. cerevisiae*.

The observed peculiarities of promoter organization are necessary but not sufficient conditions for the correct promoter activity. Whole transcriptome analysis of mammalian species revealed that approximately two-thirds of genomic DNA is randomly transcribed [57] however the transcripts are not further transformed into the high-grade protein products.

## Methods

### Profiles construction

X-axes of the profiles define the position relative to the TSS, which was denoted as +1 bp while negative and positive numbers denote upstream and downstream regions. Y-axes present the mean value of a chosen characteristic from the corresponding databases. For textual characteristics, defined at the mononucleotide level, for every 60 positions in the X-axis (numbered: -50,-49,…. -1, +1,+2,… +10) the amounts of each type of nucleotides (A, C, G, T) in all core promoters from a set of chosen species are summed up, and the resulting sum is divided on the number of the promoters. For physical or structural characteristics, defined at the base-pair step level, or for ultrasound cleavage rates at the dinucleotide level, for every 59 positions in the X-axis (numbered: -49,-48,…. -1, +1,+2,… +10) the values of these characteristics are summed up (for dinucleotides at the positions [(-50,-49); (-49,-48); …(-1,+1); …(+9,+10)], taken from DiPro DB or Additional file 13: Table S1 and the resulting sum is divided on the number of the promoters. For ultrasound cleavage rates at the tetranucleotide level, for every 57 positions in X-axis (numbered: -48,-47,…. -1, +1,+2,… +9) the values of these characteristics for tetranucleotides are summed up (for tetranucleotides at the positions (-50,-49,-48,-47); (-49,-48,-47,-46); …(-2,-1,+1,+2); …(+7,+8,+9,+10)), taken from Additional file 14: Table S2, and the resulting sum is divided on the number of the promoters. For DNAse cleavage rates at hexanucleotide level, for every 55 positions in the X-axis (numbered: -47,-46,…. -1, +1,+2,… +7,+8) the values of these characteristics are summed up (for hexanucleotides at the positions (-50,-49,-48,-47,-46,-45); (-49,-48,-47,-46,-45,-44); …(-3,-2,-1,+1,+2,+3); …(+5,+6,+7,+8,+9,+10)), taken from Supplementary to work [33]) and the resulting sum is divided on the number of the promoters.

## Additional files

Additional file 1: Show logo-representation of the core promoter sequences for six metazoan species. (PDF 647 kb)
Additional file 2: Show logo-representations of the core promoter sequences in *S. cerevisiae* and *S. pombe*. (PDF 823 kb)
Additional file 3:
**a** show distributions of dinucleotides (in percentages) in the core promoter sequences of *H. sapiens*. **b** show distributions of dinucleotides (in percentages) in the core promoter sequences of *M.musculus* and *D. melanogaster*. **c** show distributions of dinucleotides (in percentages) in the core promoter sequences of *C. elegans*. **d** show distributions of dinucleotides (in percentages) in the core promoter sequences of *D. rerio*. **e** show distributions of dinucleotides (in percentages) in the core promoter sequences of *A. thaliana* and *S. cerevisiae*. **f** show distributions of dinucleotides (in percentages) in the core promoter sequences of *S. pombe*. (PDF 6776 kb)
Additional file 4:
**a** show frequencies of tetranucleotide occurrences (in percentages) in terms of “Py,Pu” in *H. sapiens* and *M. musculus*. **b** show frequencies of tetranucleotide occurrences (in percentages) in terms of “Py,Pu” in *D. melanogaster* and *C. elegans*. **c** show frequencies of tetranucleotide occurrences (in percentages) in terms of “Py,Pu” in *D. rerio* and *A. thaliana*. **d** show frequencies of tetranucleotide occurrences (in percentages) in terms of “Py,Pu” in *S. cerevisiae*. **e** show frequencies of tetranucleotide occurrences (in percentages) in terms of “Py,Pu” in *S. pombe*. (PDF 6997 kb)
Additional file 5: Archive S1. (excel-spreadsheets with the data on tetranucleotides distribution in “PyPu” description). (RAR 178 kb)
Additional file 6: Show entropy variations for the set of core promoters of *H. sapiens,* calculated using two variants of parametrization [33, 34]. (PDF 32 kb)
Additional file 7: (A-H) show the ultrasonic and DNase I cleavage profiles for both complementary strands in the core promoters of *A. thaliana*. (PDF 270 kb)
Additional file 8: (A-H) show the ultrasonic and DNase I cleavage profiles for both complementary strands in the core promoters of *D. melanogaster*. (PDF 273 kb)
Additional file 9: (A-H) show the ultrasonic and DNase I cleavage profiles for both complementary strands in the core promoters of *C. elegans*.(PDF 276 kb)
Additional file 10: (A-H) show the ultrasonic and DNase I cleavage profiles for both complementary strands in the core promoters of *D. rerio*. (PDF 284 kb)
Additional file 11: (A-H) show the ultrasonic and DNase I cleavage profiles for both complementary strands in the core promoters of *M. musculus*. (PDF 275 kb)
Additional file 12: Archive S2. (excel-spreadsheets with profiles of ultrasound cleavage indexes ***T*** and ***S*** in the subsets of promoters which contain and does not contain TATA-tetranucleotide for all species analysed.) (RAR 331 kb)
Additional file 13:Table S1. is the DataBase for ultrasonic cleavage rates at the dinucleotide level of resolution [23]. (DOCX 17 kb)
Additional file 14: Table S2. is the DataBase for ultrasonic cleavage at the tetranucleotide level of resolution [23]. (DOCX 67 kb)

## Abbreviations

bp: Base pair
DNase I: Bovine pancreatic deoxyribonuclease I
Pol II: RNA polymerase II
TBP: TATA-binding proteins
TFs: Transcription factors
TSS: Transcription start site
