# Supplementary figures and images for: Structural features of DNA that determine RNA polymerase II core promoter
Source: BMC Genomics. 2016 Nov 25;17:973. doi: 10.1186/s12864-016-3292-z (PMC5123417; doi:10.1186/s12864-016-3292-z)

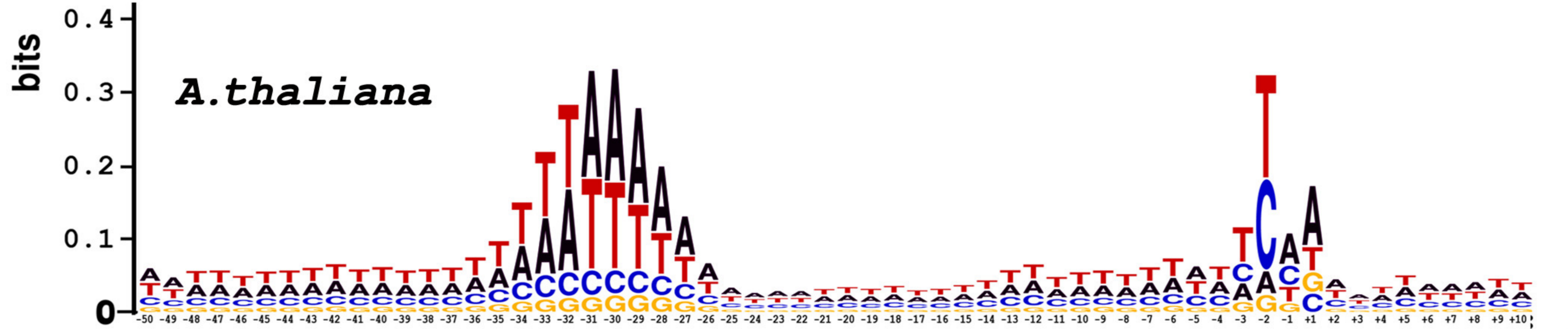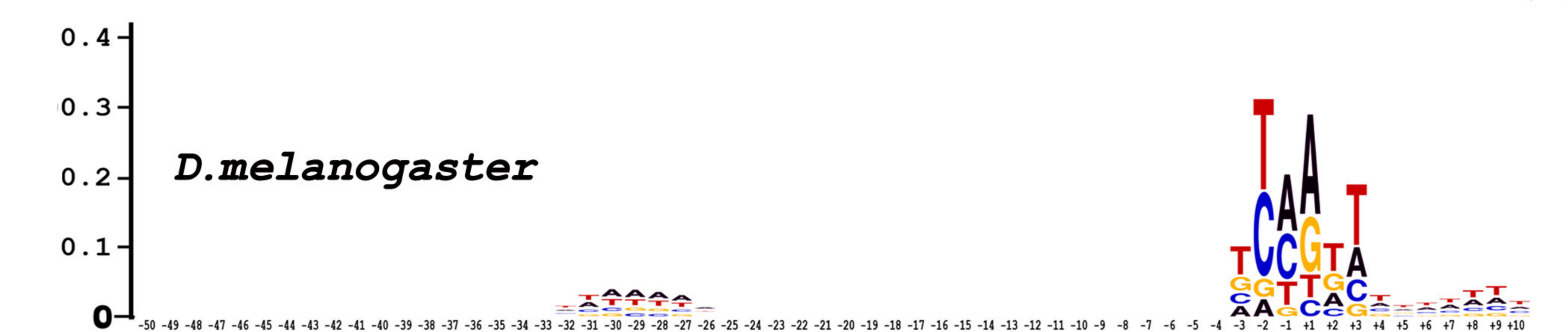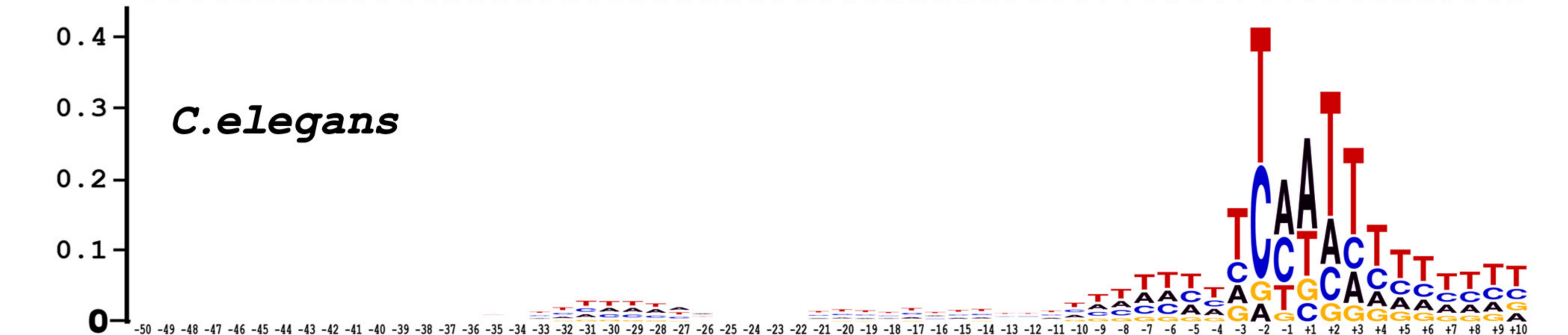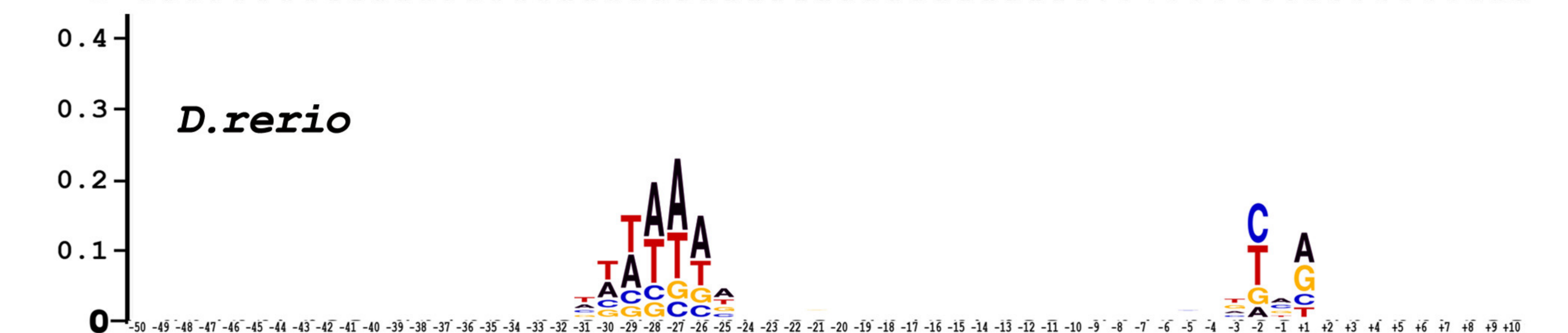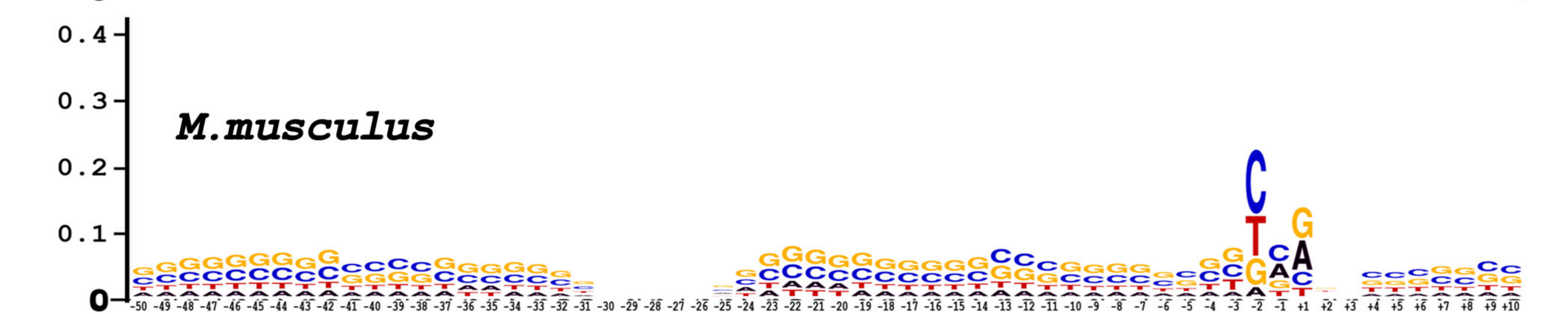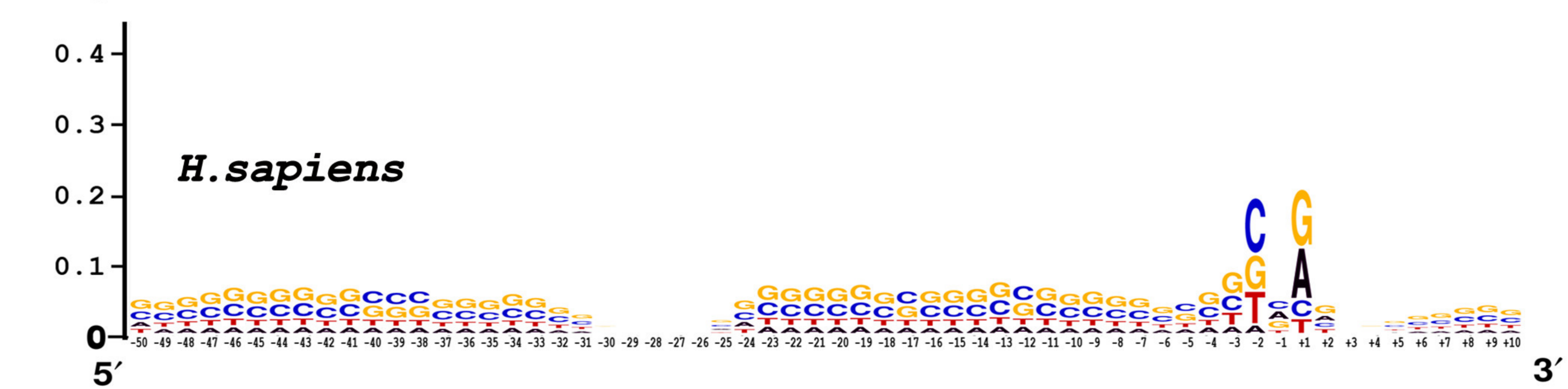

Supplement: Additional file 1: — Show logo-representation of the core promoter sequences for six metazoan species. (PDF 647 kb) [file 12864_2016_3292_MOESM1_ESM.pdf]

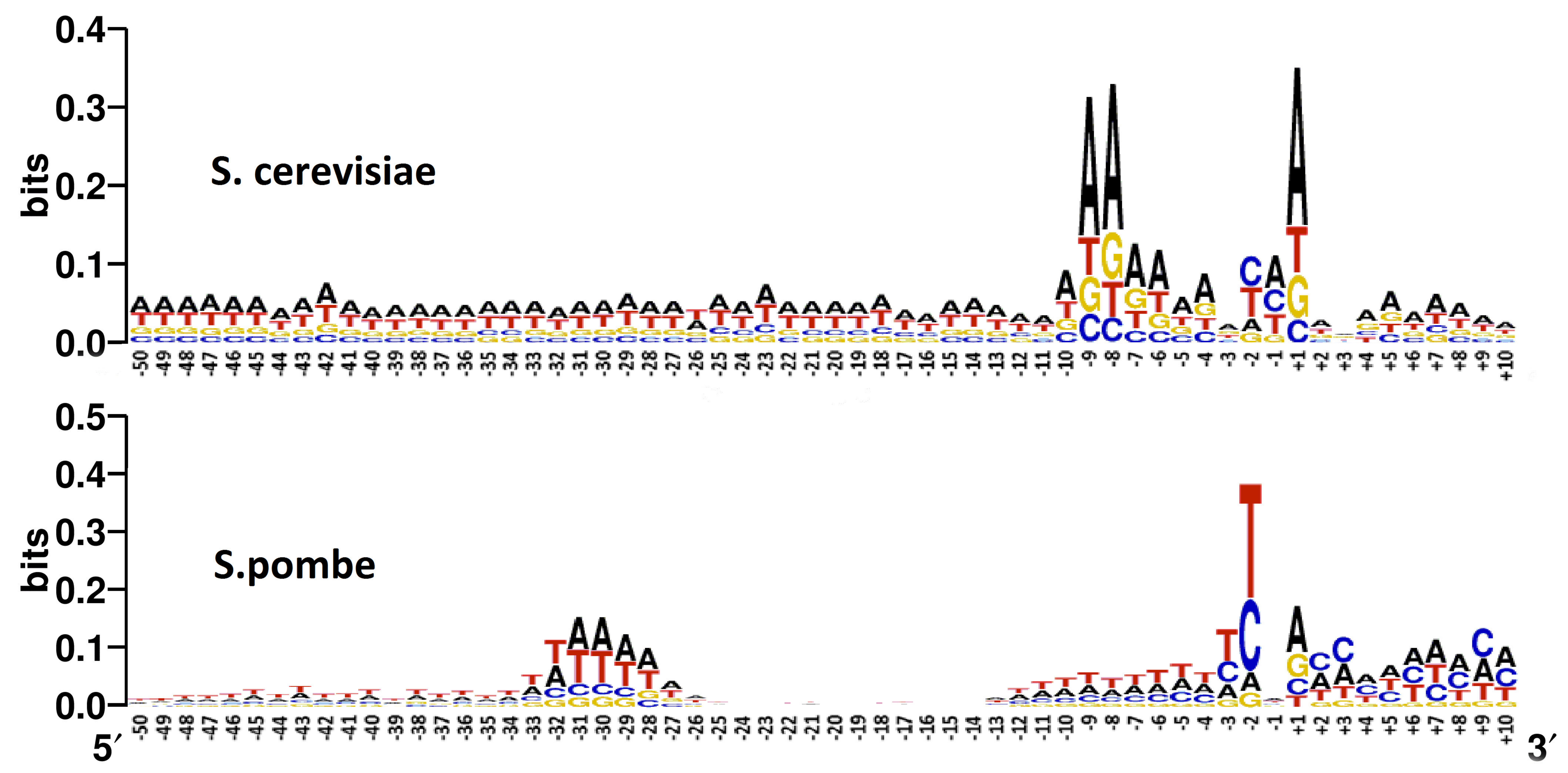

Supplement: Additional file 2: — Show logo-representations of the core promoter sequences in S. cerevisiae and S. pombe. (PDF 823 kb) [file 12864_2016_3292_MOESM2_ESM.pdf]

# *H. sapiens*

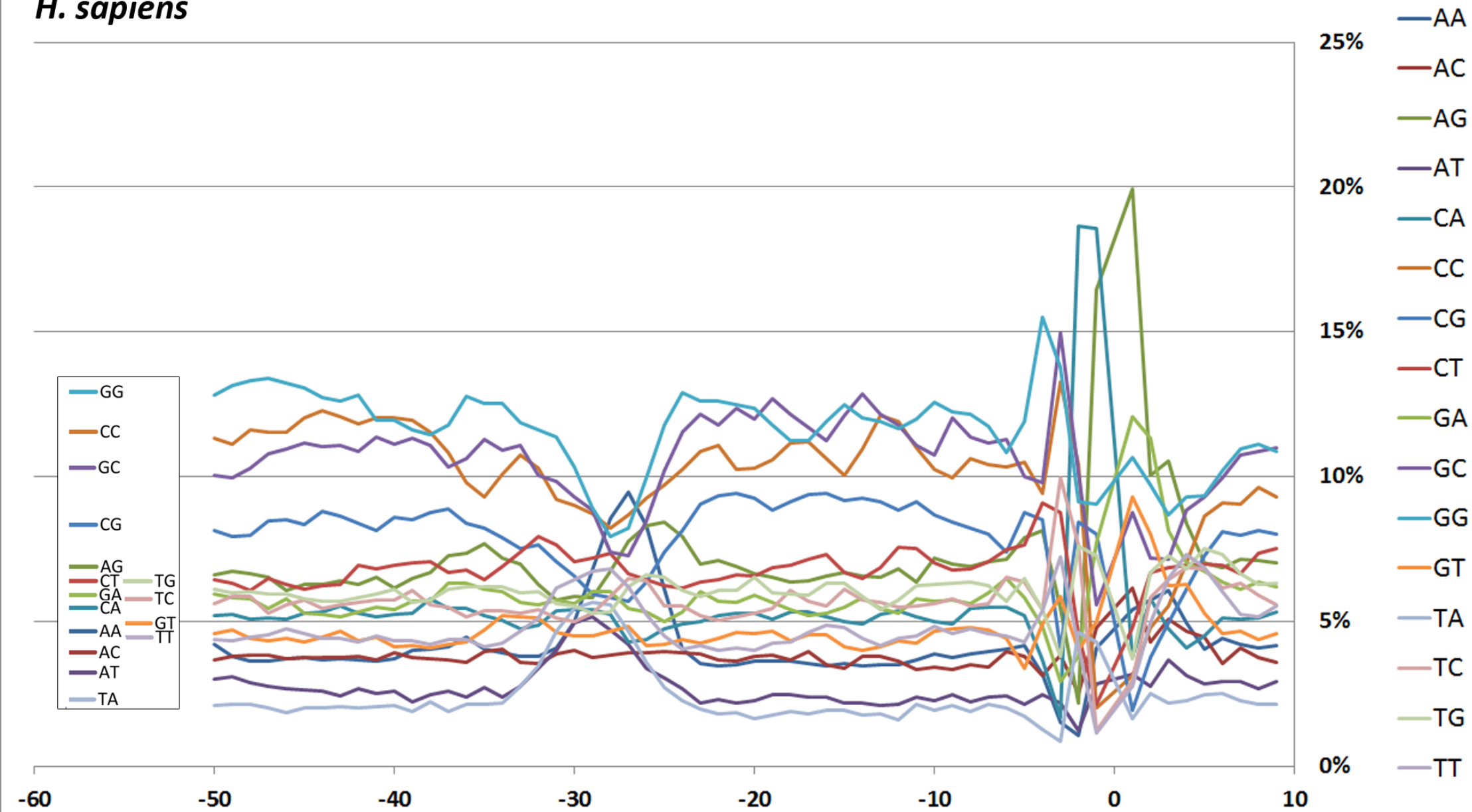

# *M. musculus*

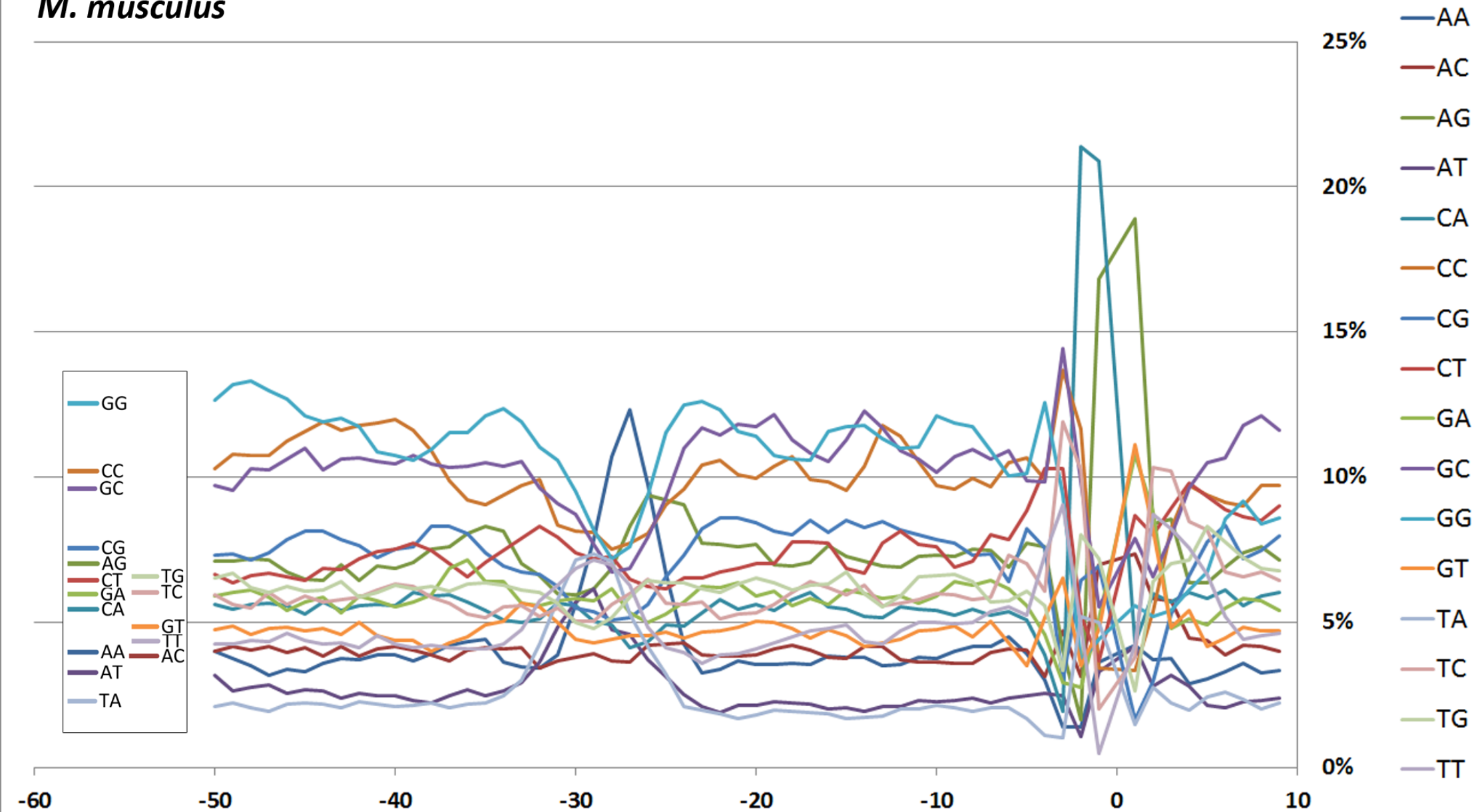

***D. melanogaster***

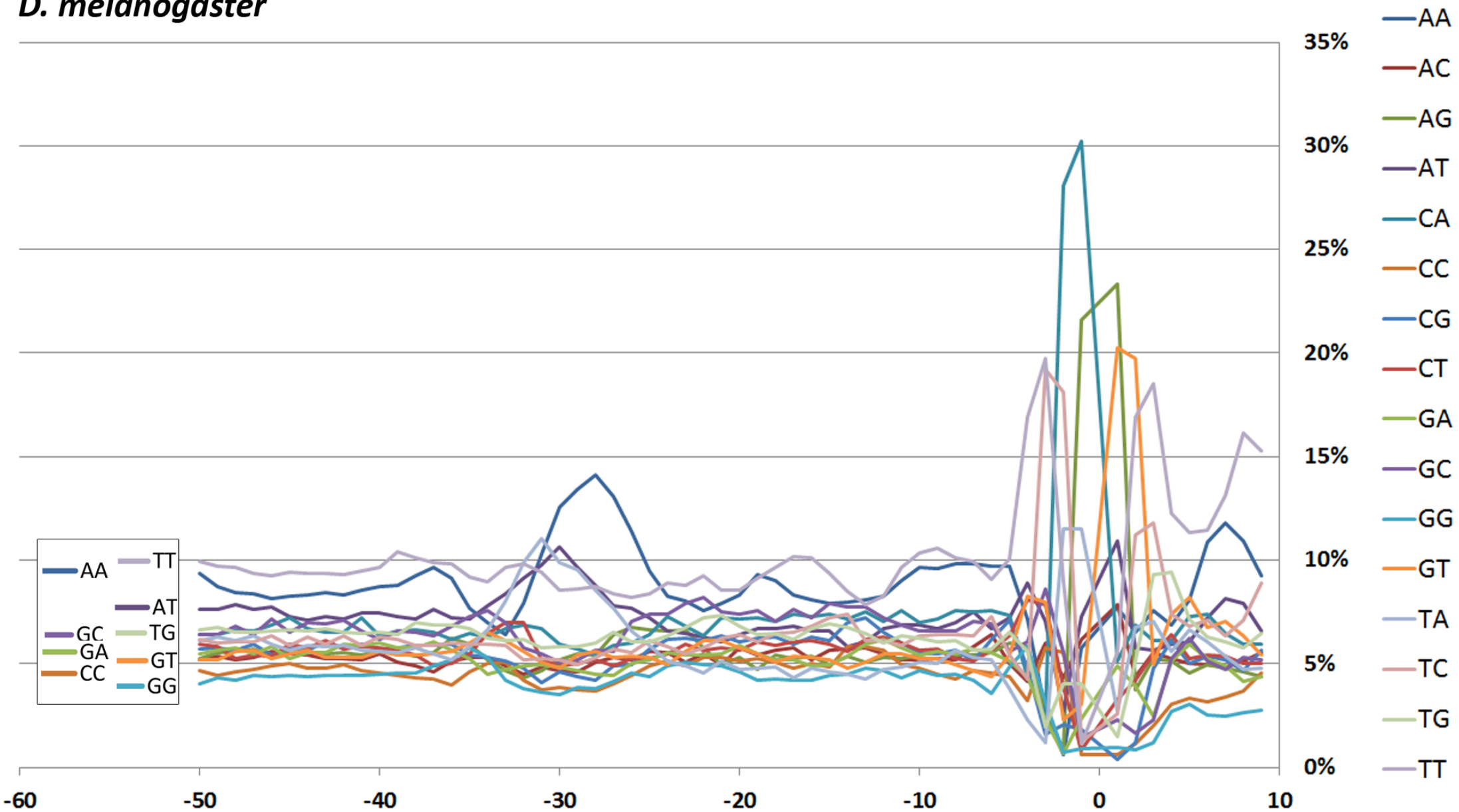

*D. rerio*

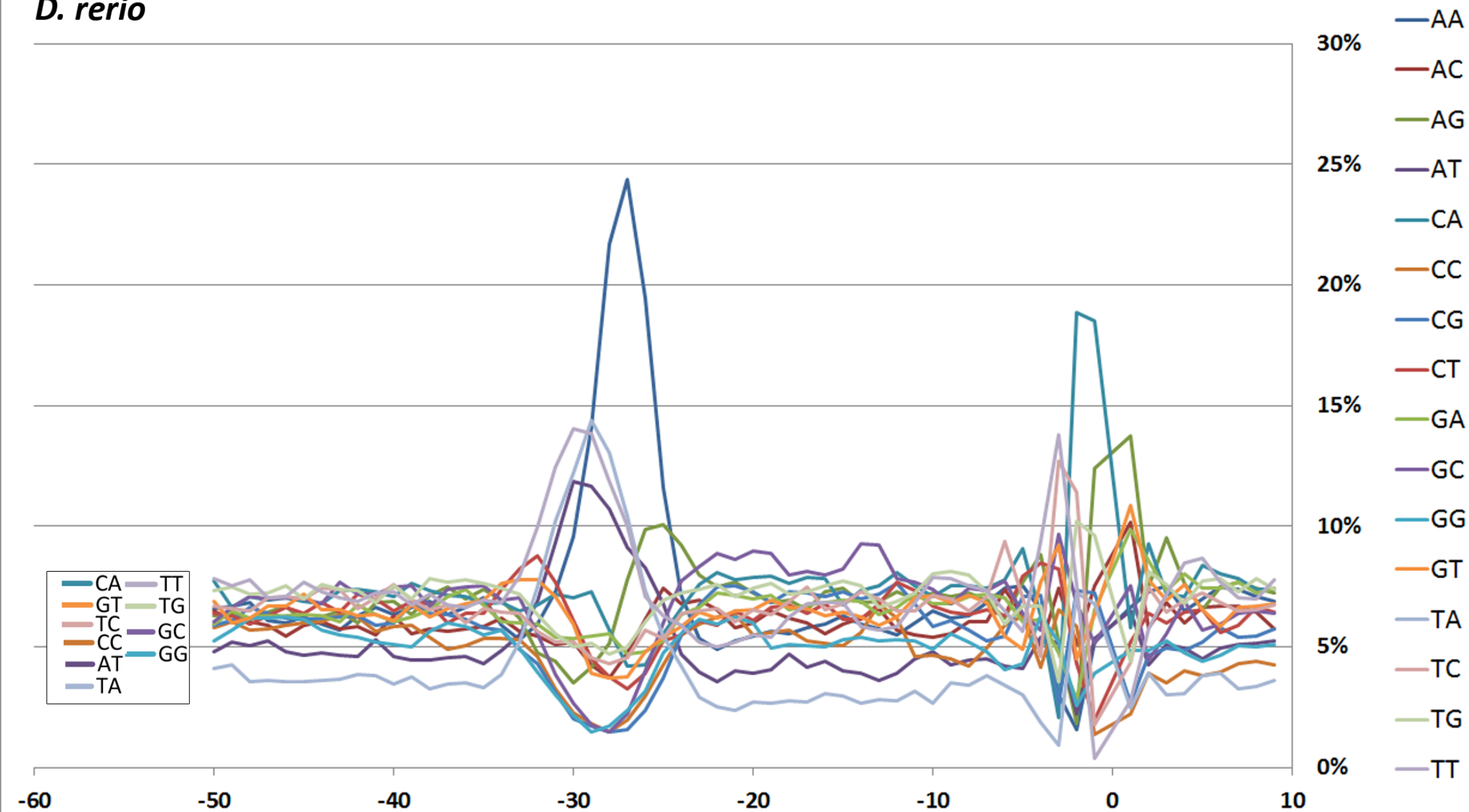

# *C. elegans*

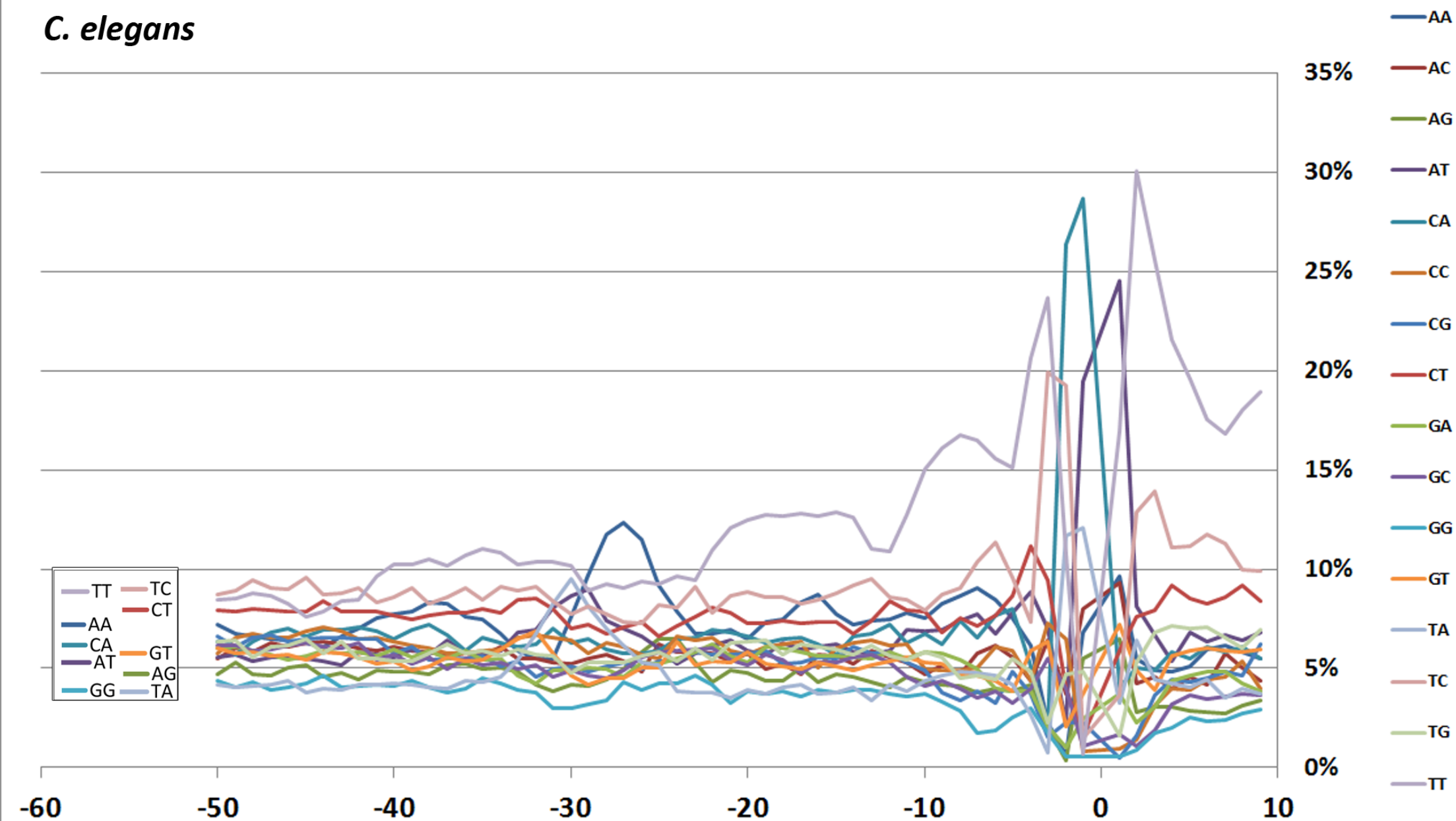

# *A. thaliana*

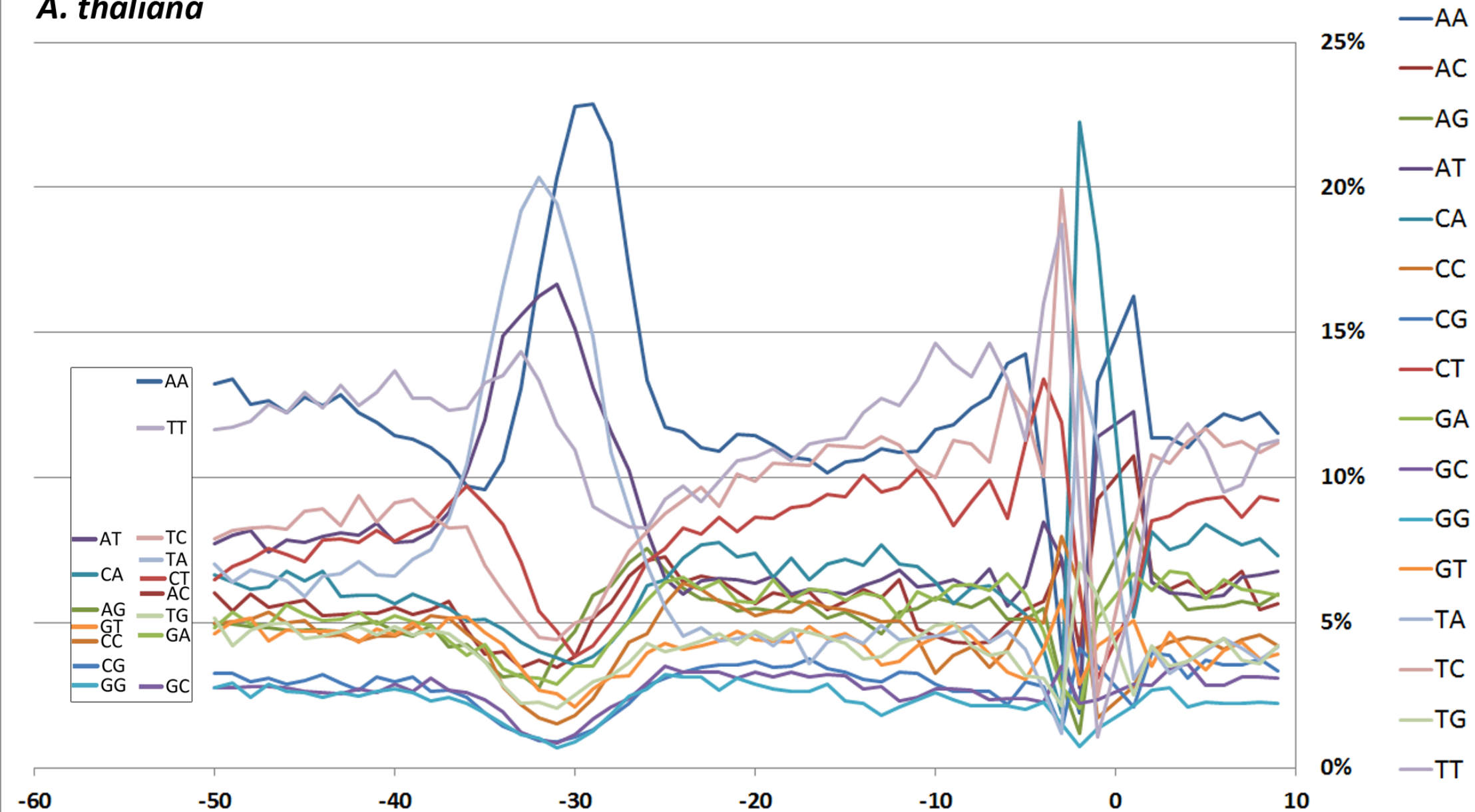

# *S. cerevisiae*

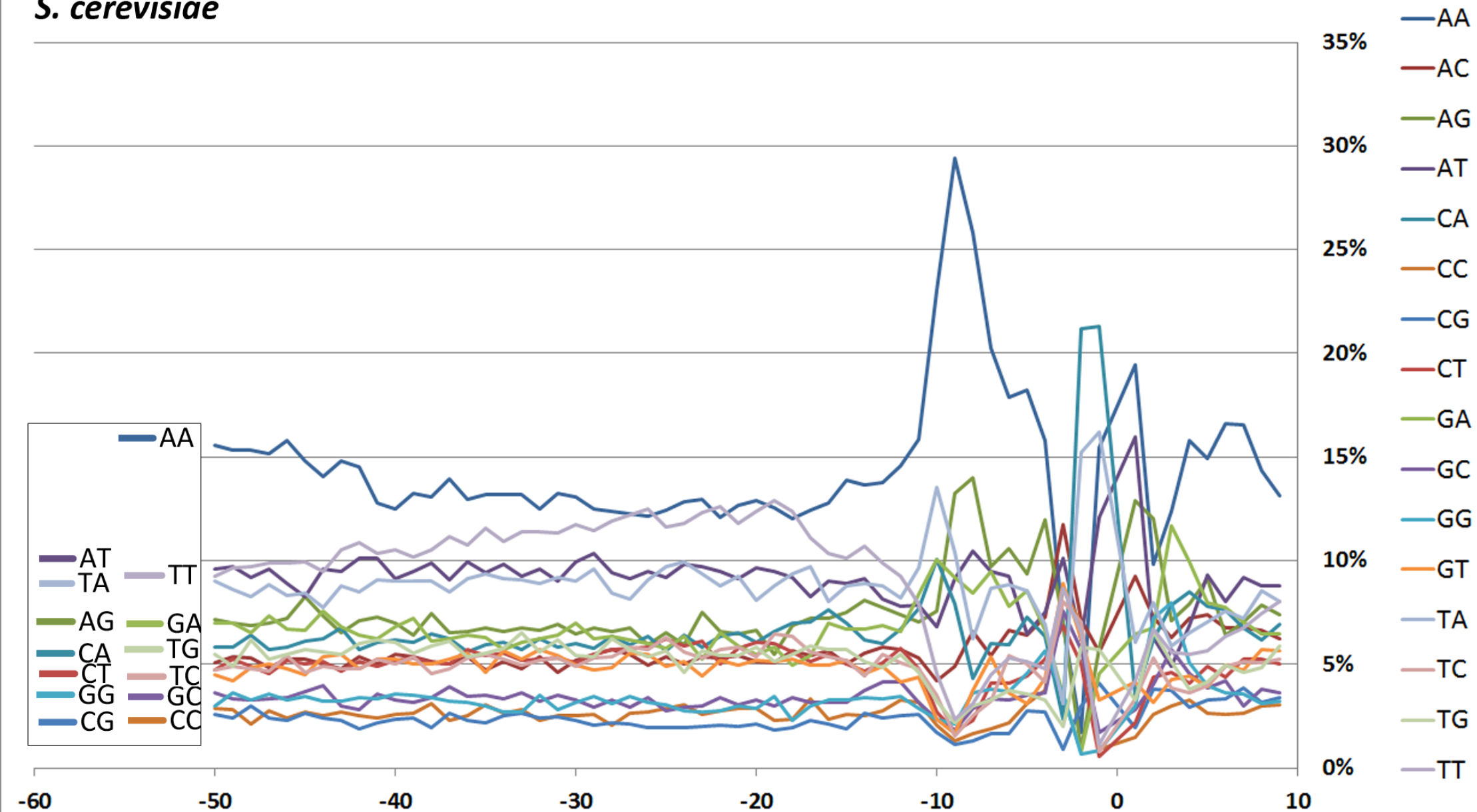

*S. pombe*

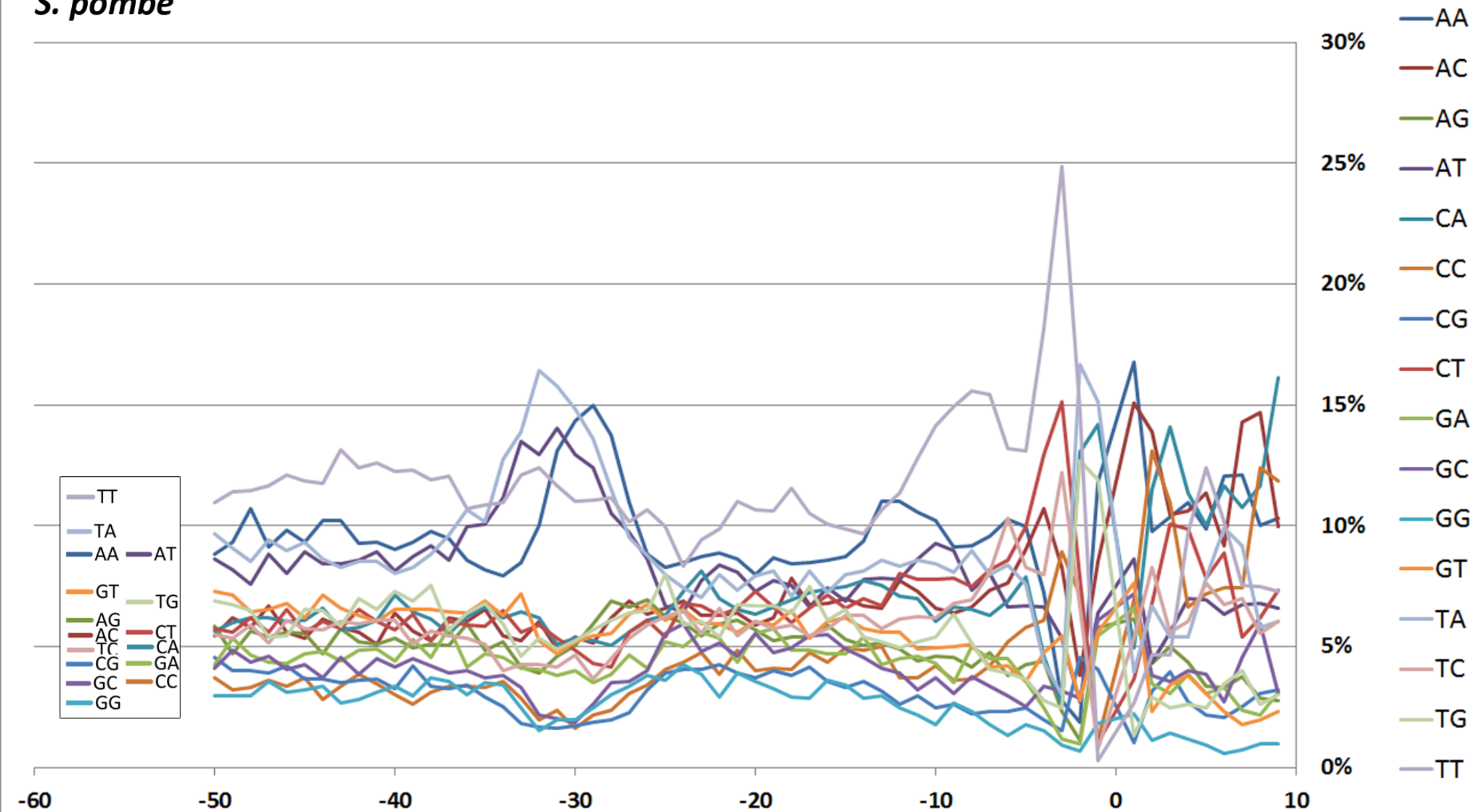

Supplement: Additional file 3: — a show distributions of dinucleotides (in percentages) in the core promoter sequences of H. sapiens. b show distributions of dinucleotides (in percentages) in the core promoter sequences of M.musculus and D. melanogaster. c show distributions of dinucleotides (in percentages) in the core promoter sequences of C. elegans. d show distributions of dinucleotides (in percentages) in the core promoter sequences of D. rerio. e show distributions of dinucleotides (in percentages) in the core promoter sequences of A. thaliana and S. cerevisiae. f show distributions of dinucleotides (in percentages) in the core promoter sequences of S. pombe. (PDF 6776 kb) [file 12864_2016_3292_MOESM3_ESM.pdf]

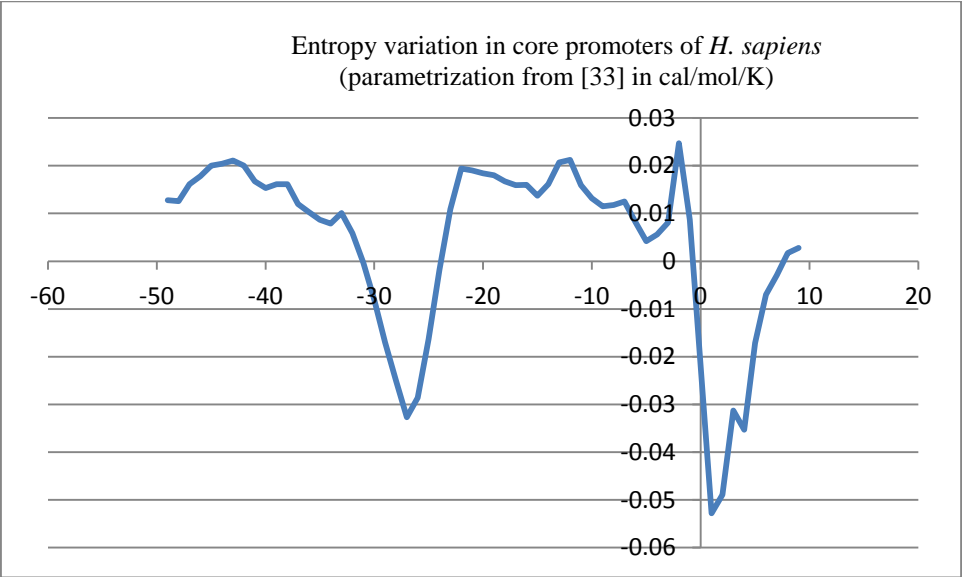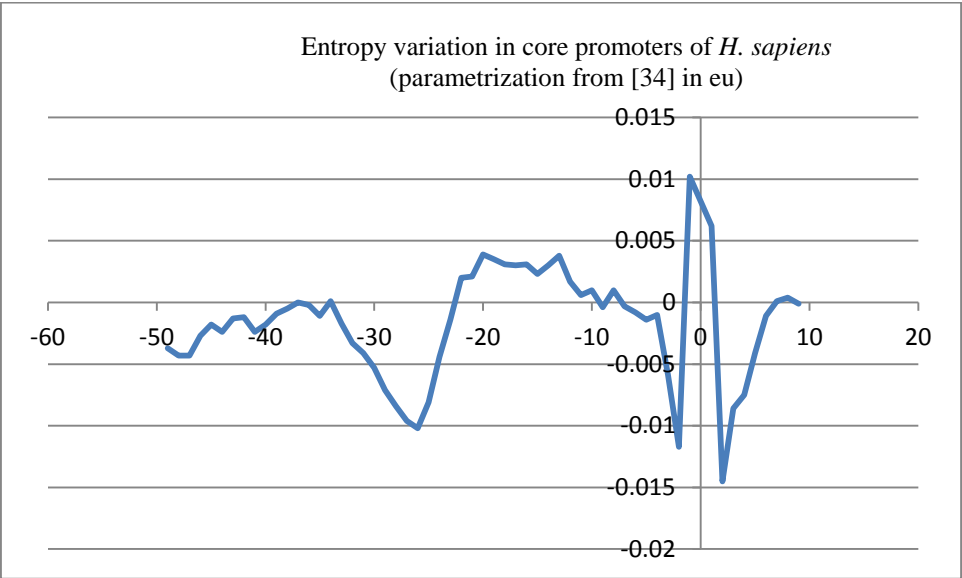

Supplement: Additional file 6: — Show entropy variations for the set of core promoters of H. sapiens, calculated using two variants of parametrization [33, 34]. (PDF 32 kb) [file 12864_2016_3292_MOESM6_ESM.pdf]

## Ultrasound indexes for *A. thaliana*

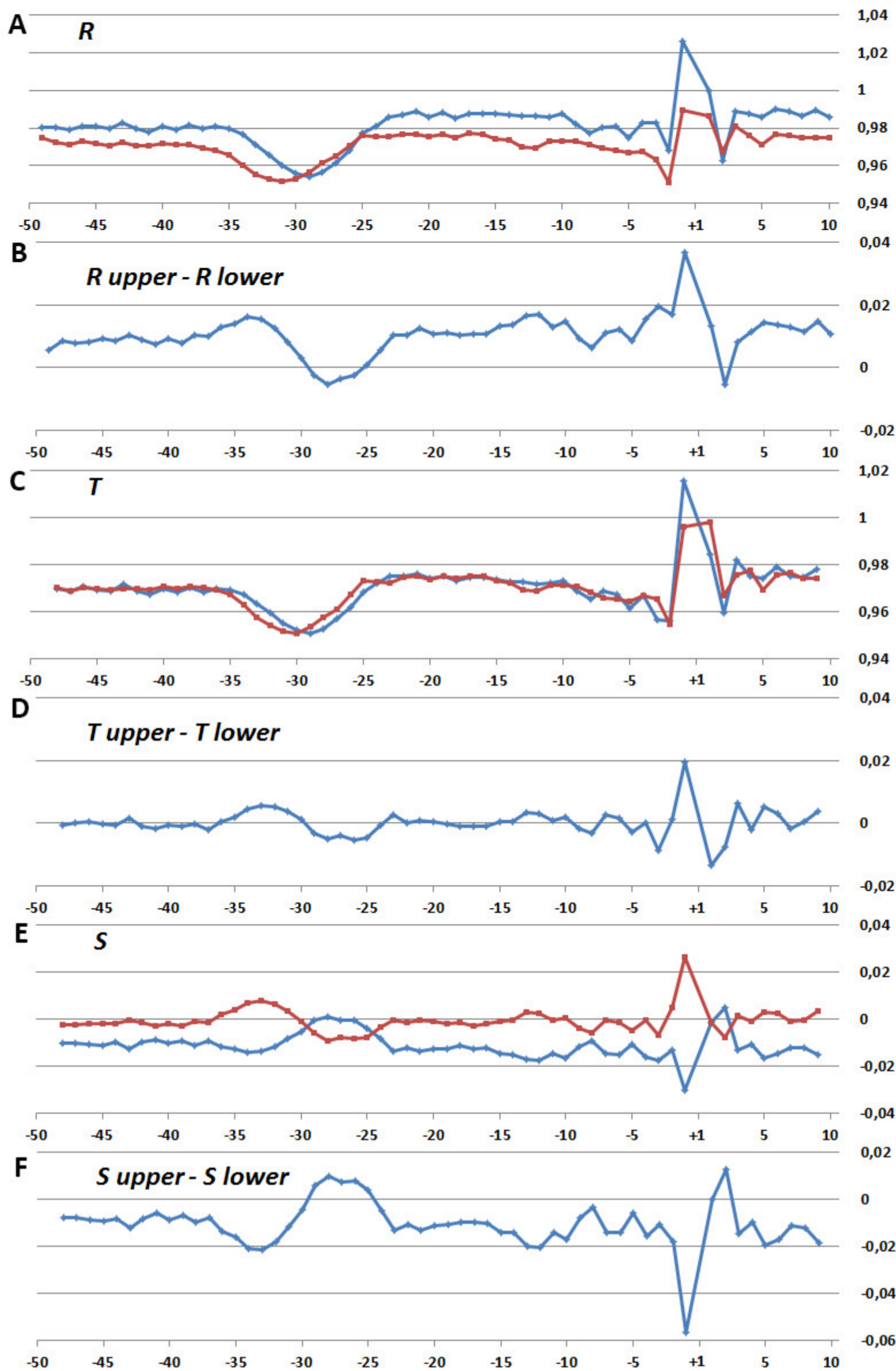

## DNase indexes for *A. thaliana*

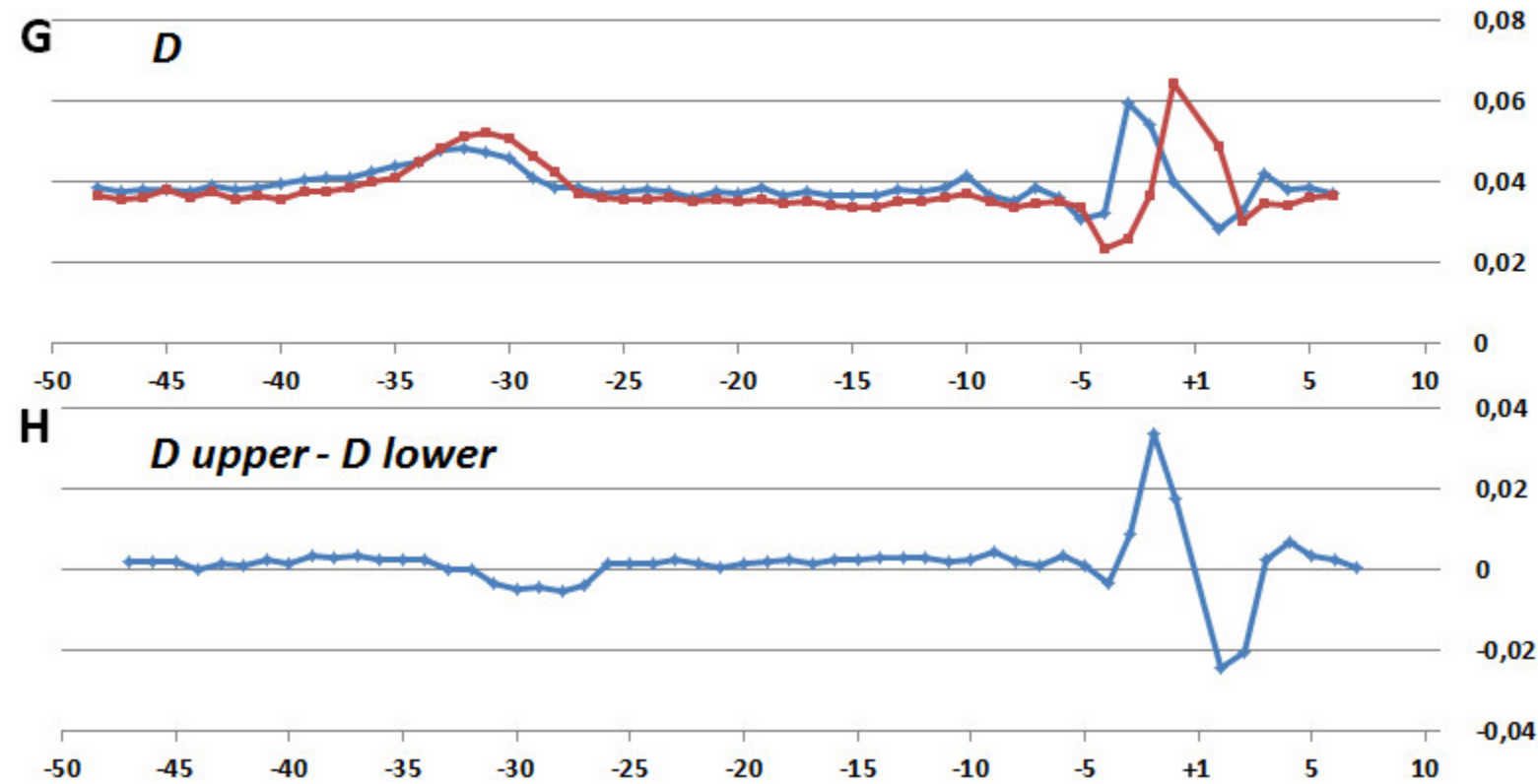

Supplement: Additional file 7: — (A-H) show the ultrasonic and DNase I cleavage profiles for both complementary strands in the core promoters of A. thaliana. (PDF 270 kb) [file 12864_2016_3292_MOESM7_ESM.pdf]

## Ultrasound indexes for *D. melanogaster*

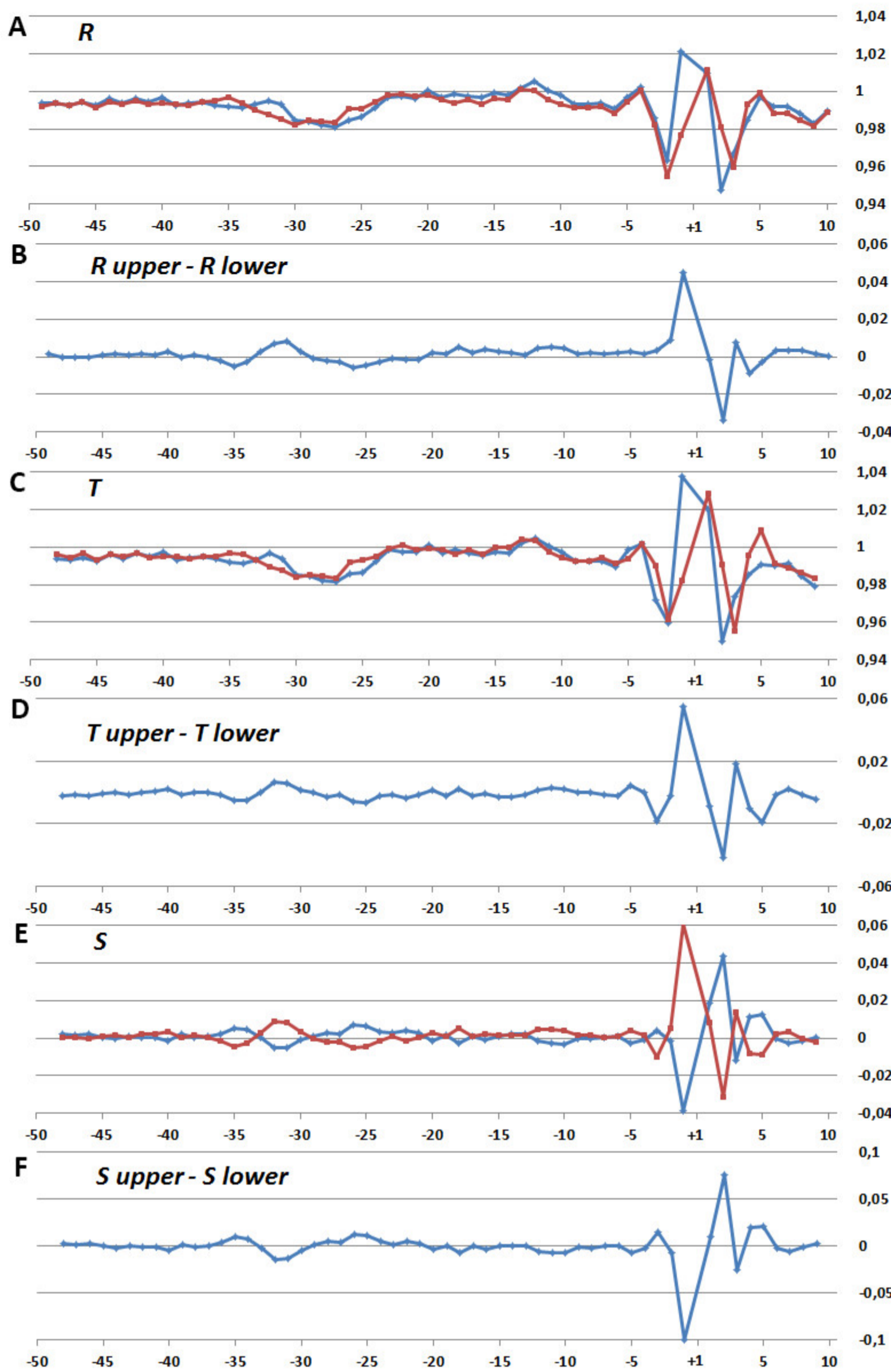

## DNase indexes for *D. melanogaster*

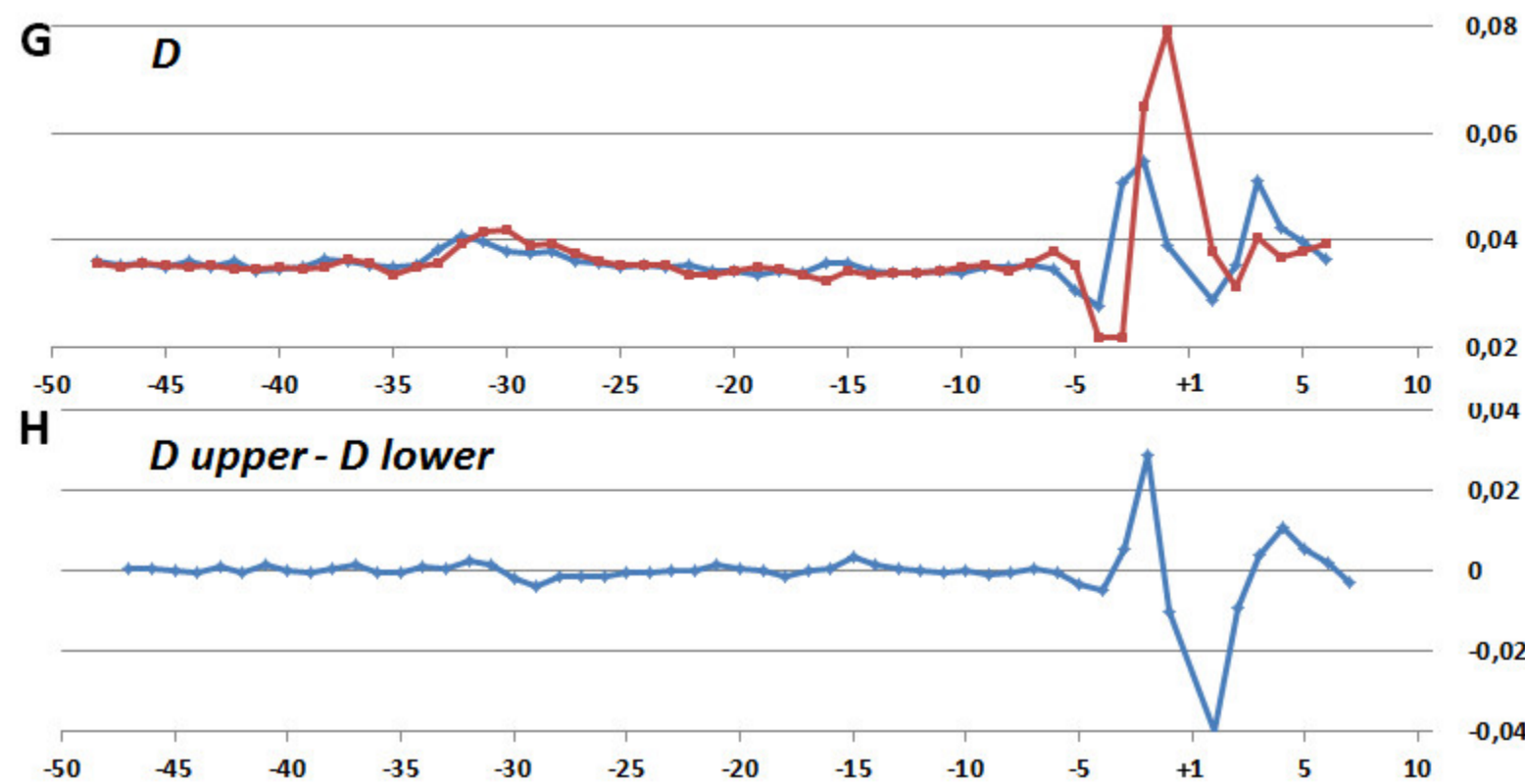

Supplement: Additional file 8: — (A-H) show the ultrasonic and DNase I cleavage profiles for both complementary strands in the core promoters of D. melanogaster. (PDF 273 kb) [file 12864_2016_3292_MOESM8_ESM.pdf]

## Ultrasound indexes for *C.elegans*

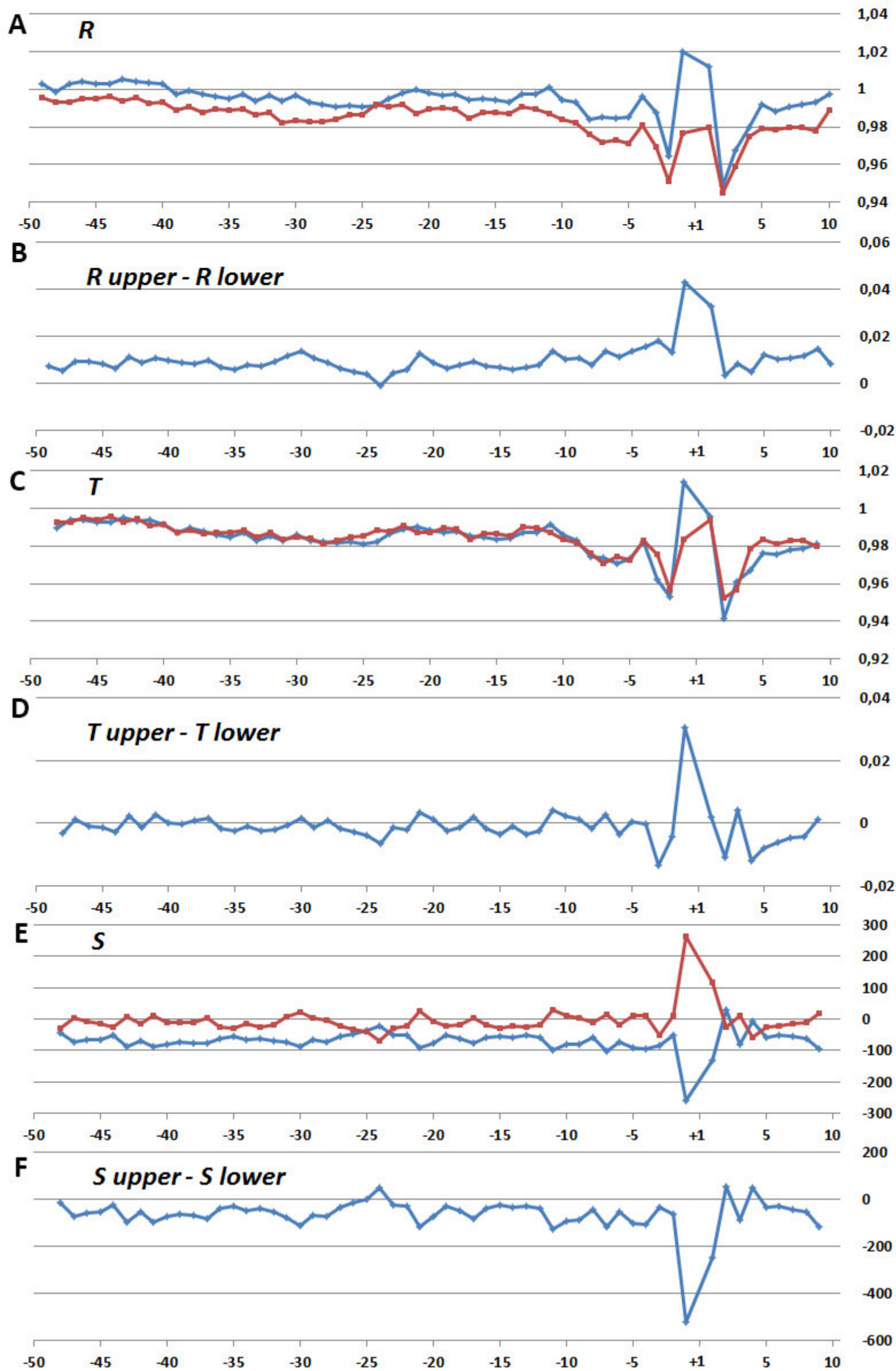

## DNAse indexes for *C.elegans*

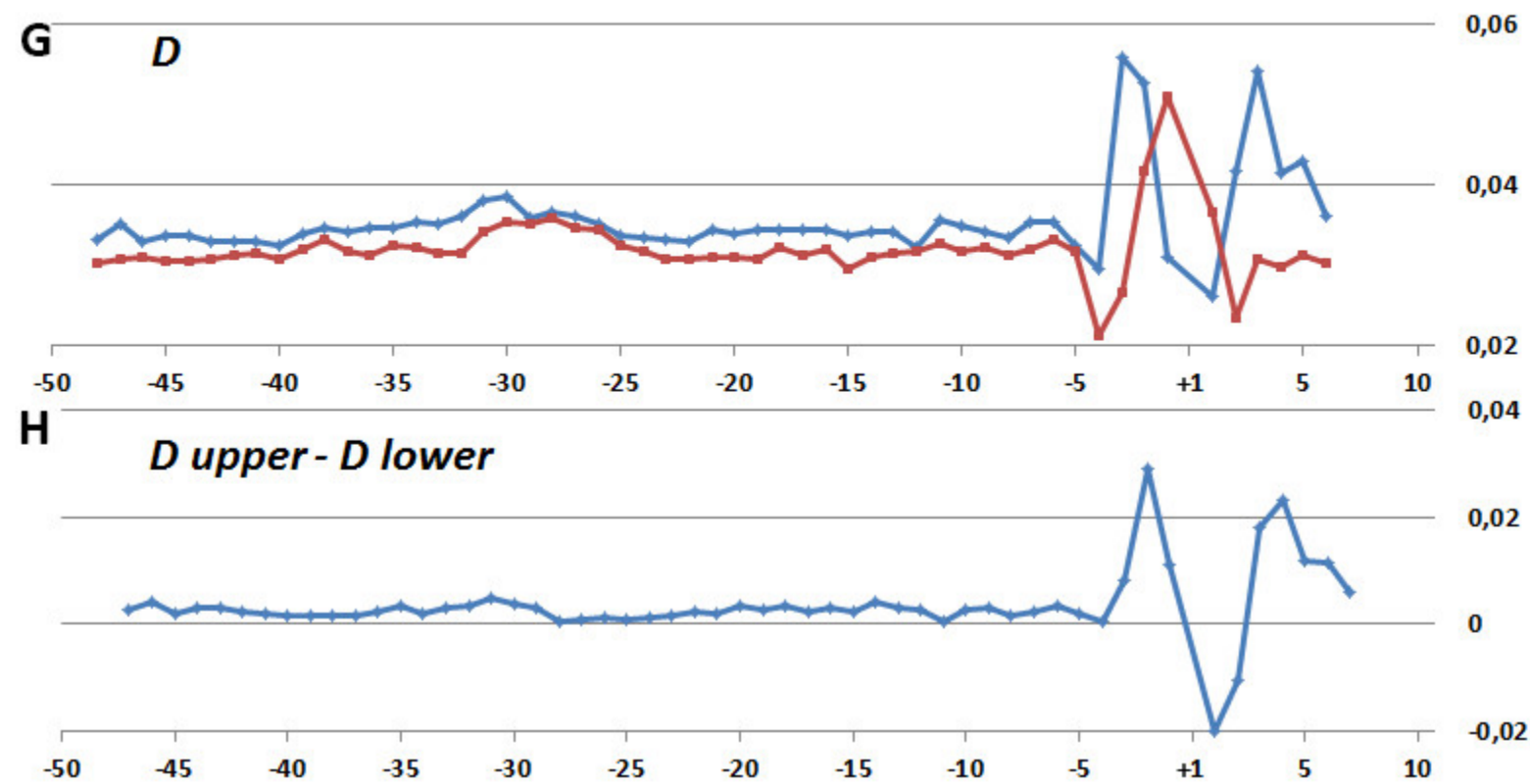

Supplement: Additional file 9: — (A-H) show the ultrasonic and DNase I cleavage profiles for both complementary strands in the core promoters of C. elegans.(PDF 276 kb) [file 12864_2016_3292_MOESM9_ESM.pdf]

## Ultrasound indexes for *D. rerio*

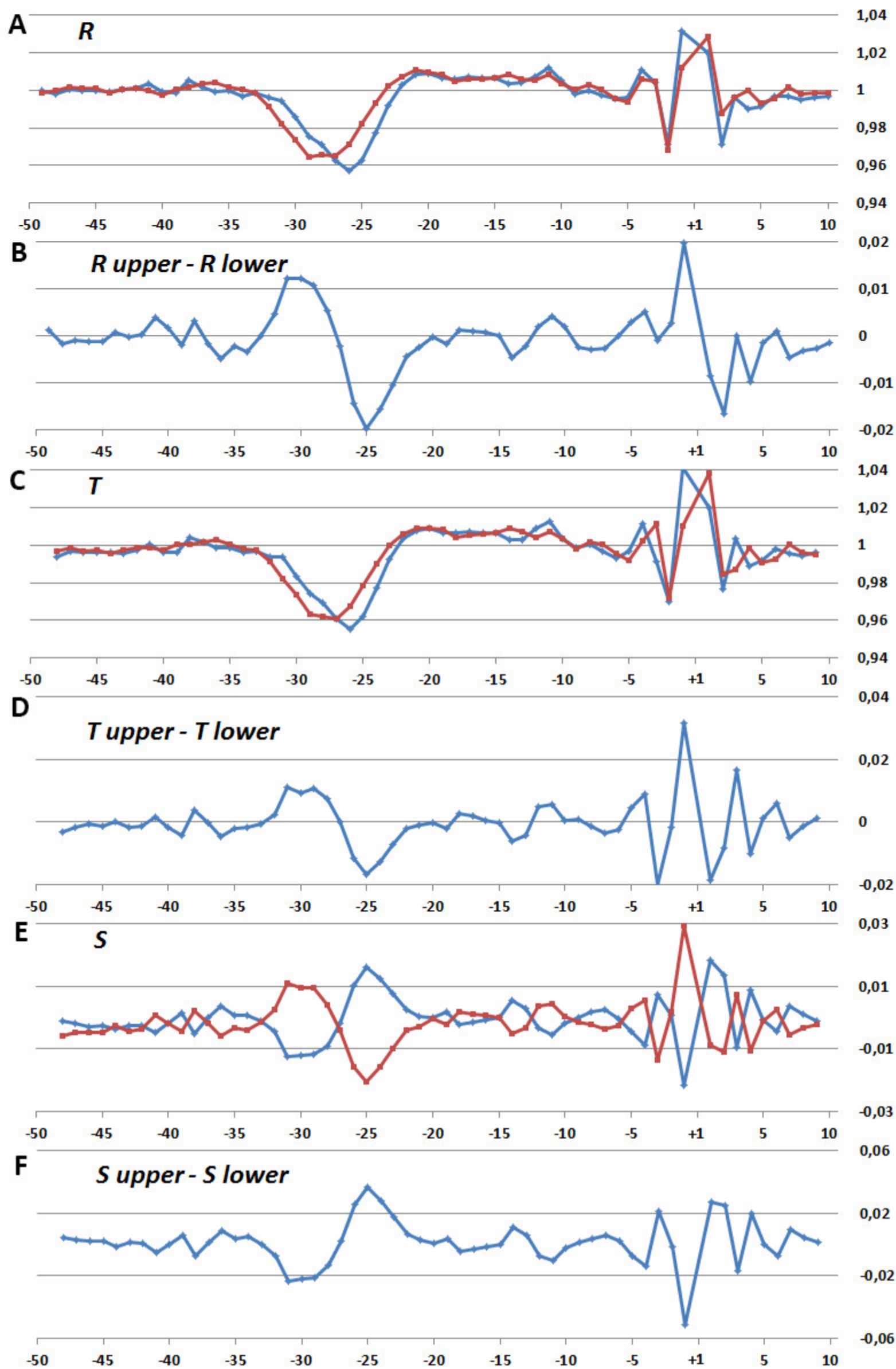

## DNase indexes for *D. rerio*

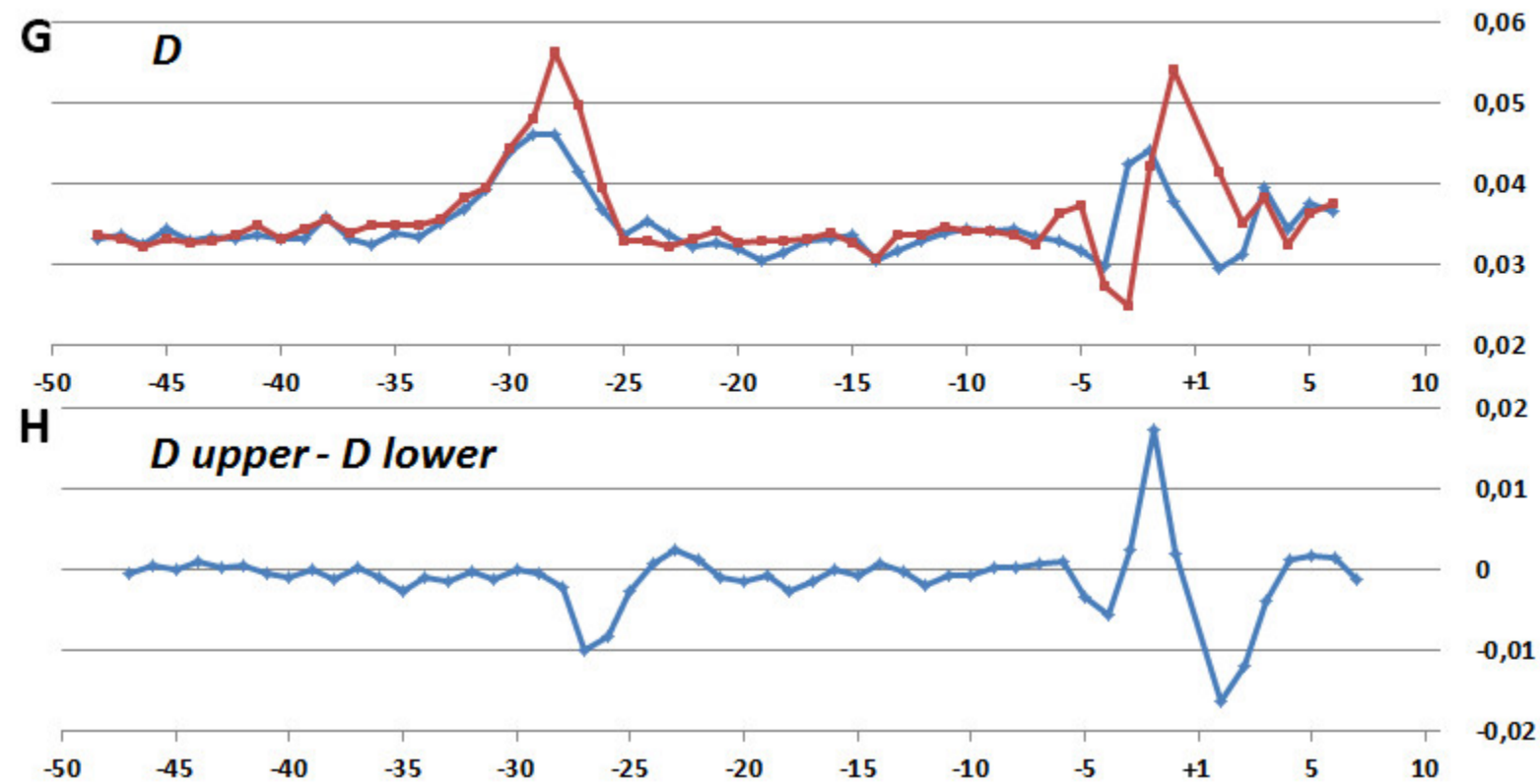

Supplement: Additional file 10: — (A-H) show the ultrasonic and DNase I cleavage profiles for both complementary strands in the core promoters of D. rerio. (PDF 284 kb) [file 12864_2016_3292_MOESM10_ESM.pdf]

## Ultrasound indexes for *M. musculus*

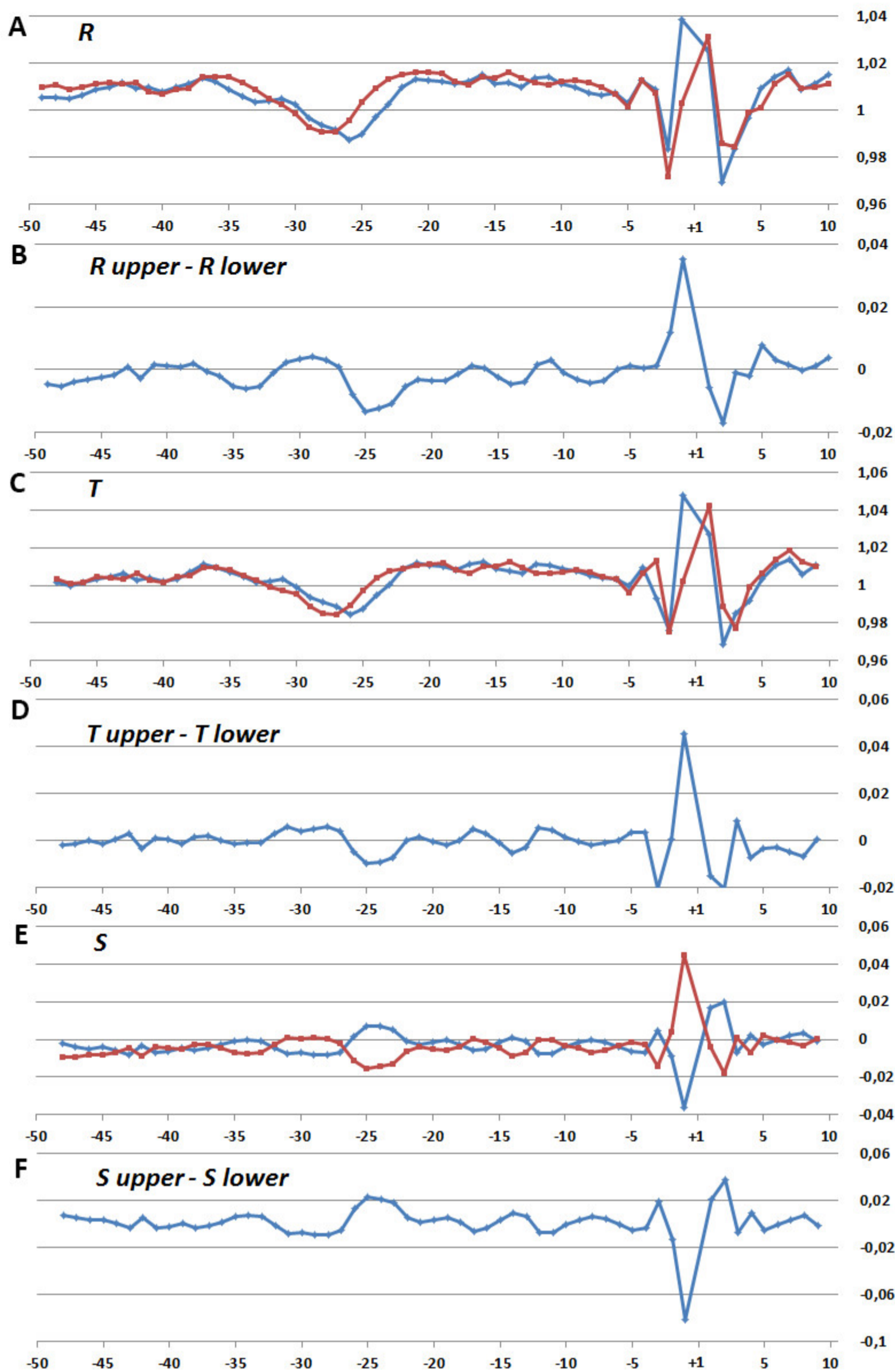

## DNase indexes for *M. musculus*

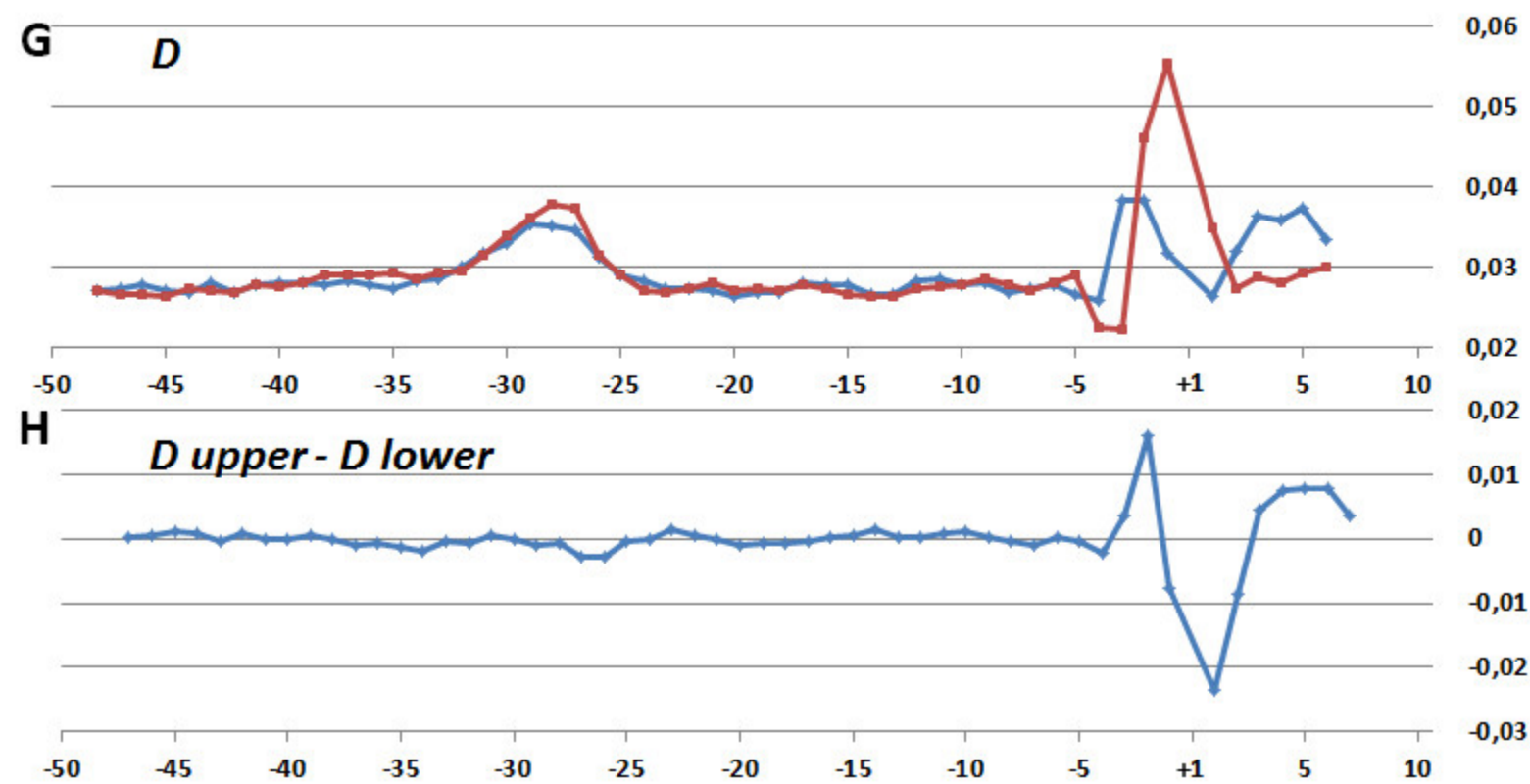

Supplement: Additional file 11: — (A-H) show the ultrasonic and DNase I cleavage profiles for both complementary strands in the core promoters of M. musculus. (PDF 275 kb) [file 12864_2016_3292_MOESM11_ESM.pdf]
