# Supplementary material for: Structural features of DNA that determine RNA polymerase II core promoter
Source: BMC Genomics. 2016 Nov 25;17:973. doi: 10.1186/s12864-016-3292-z (PMC5123417; doi:10.1186/s12864-016-3292-z)
Supplement: Additional file 4: — a show frequencies of tetranucleotide occurrences (in percentages) in terms of “Py,Pu” in H. sapiens and M. musculus. b show frequencies of tetranucleotide occurrences (in percentages) in terms of “Py,Pu” in D. melanogaster and C. elegans. c show frequencies of tetranucleotide occurrences (in percentages) in terms of “Py,Pu” in D. rerio and A. thaliana. d show frequencies of tetranucleotide occurrences (in percentages) in terms of “Py,Pu” in S. cerevisiae. e show frequencies of tetranucleotide occurrences (in percentages) in terms of “Py,Pu” in S. pombe. (PDF 6997 kb) [file 12864_2016_3292_MOESM4_ESM.pdf]

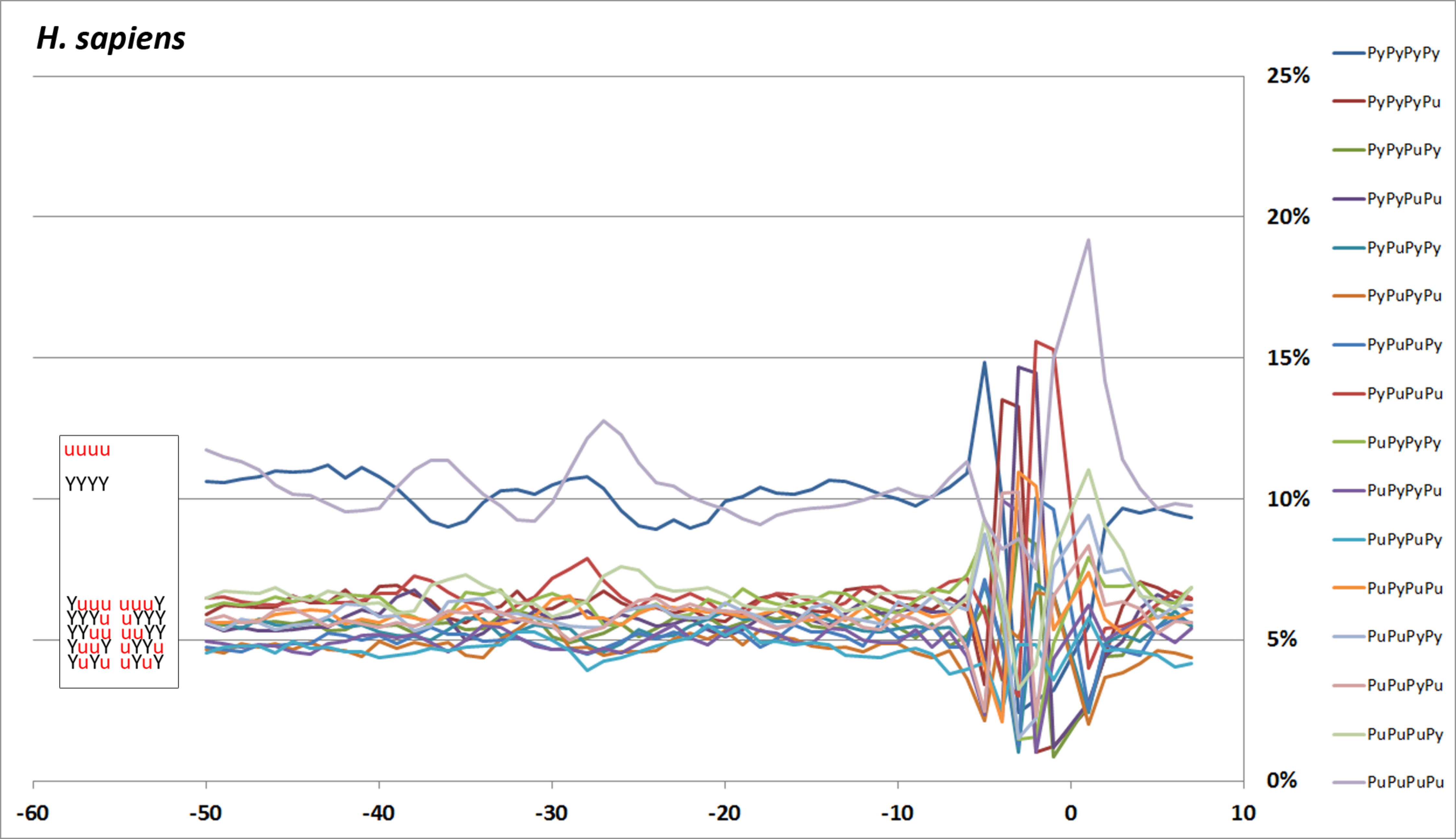

H. sapiens
uuuu
YYYY
Yuuu uuuY
YYYu uYYY
YYuu uuYY
YuuY uYYu
YuYu uYuY
25%
20%
15%
10%
5%
0%
-60
-50
-40
-30
-20
-10
0
10
PyPyPyPy
PyPyPyPu
PyPyPuPy
PyPyPuPu
PyPuPyPy
PyPuPyPu
PyPuPuPy
PyPuPuPu
PuPyPyPy
PuPyPyPu
PuPyPuPy
PuPyPuPu
PuPuPyPy
PuPuPyPu
PuPuPuPy
PuPuPuPu

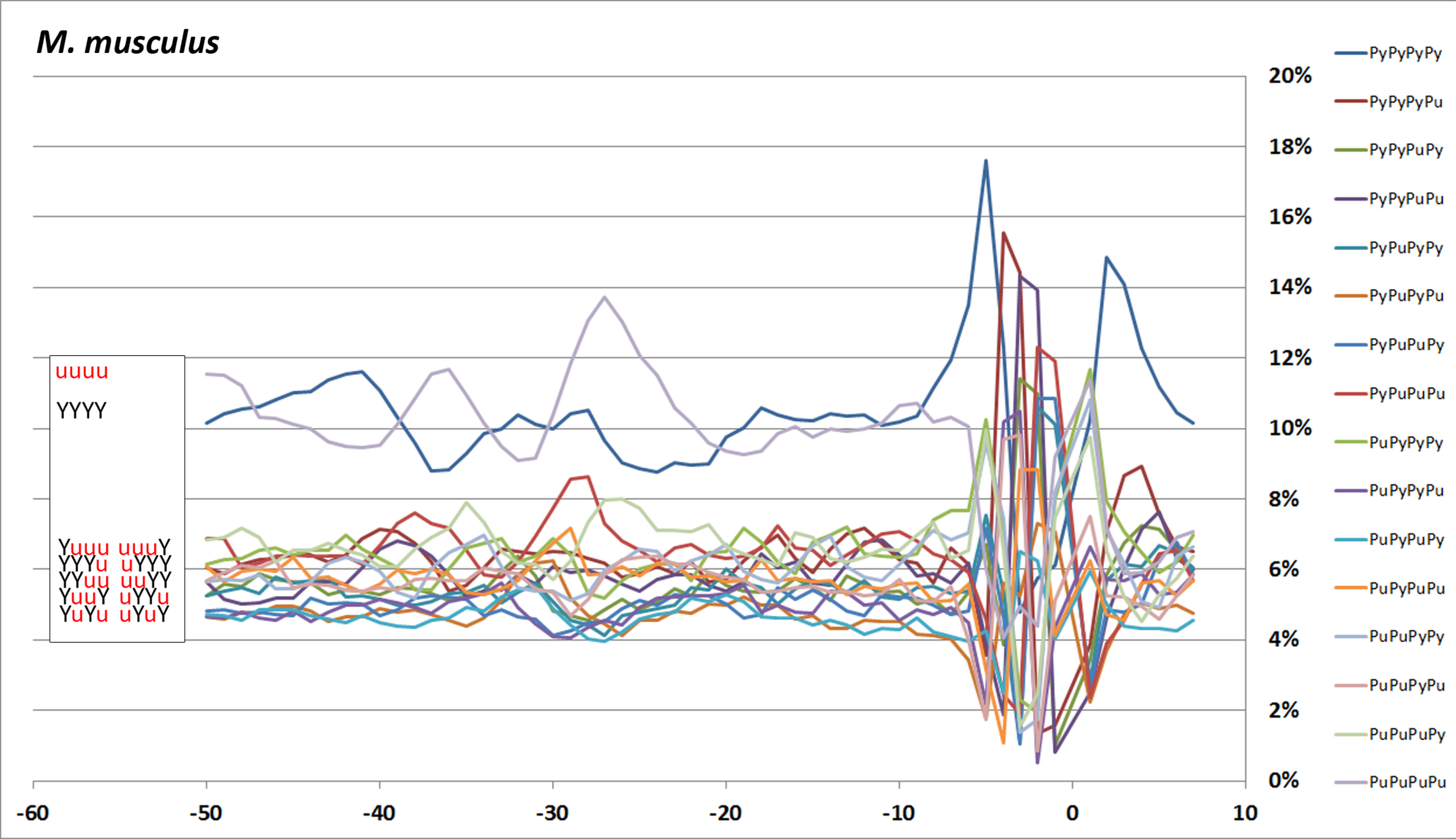
M. musculus
uuuu
YYYY
Yuuu uuuY
YYYu uYYY
YYuu uuYY
YuuY uYYu
YuYu uYuY
20%
18%
16%
14%
12%
10%
8%
6%
4%
2%
0%
-60
-50
-40
-30
-20
-10
0
10
PyPyPyPy
PyPyPyPu
PyPyPuPy
PyPyPuPu
PyPuPyPy
PyPuPyPu
PyPuPuPy
PyPuPuPu
PuPyPyPy
PuPyPyPu
PuPyPuPy
PuPyPuPu
PuPuPyPy
PuPuPyPu
PuPuPuPy
PuPuPuPu

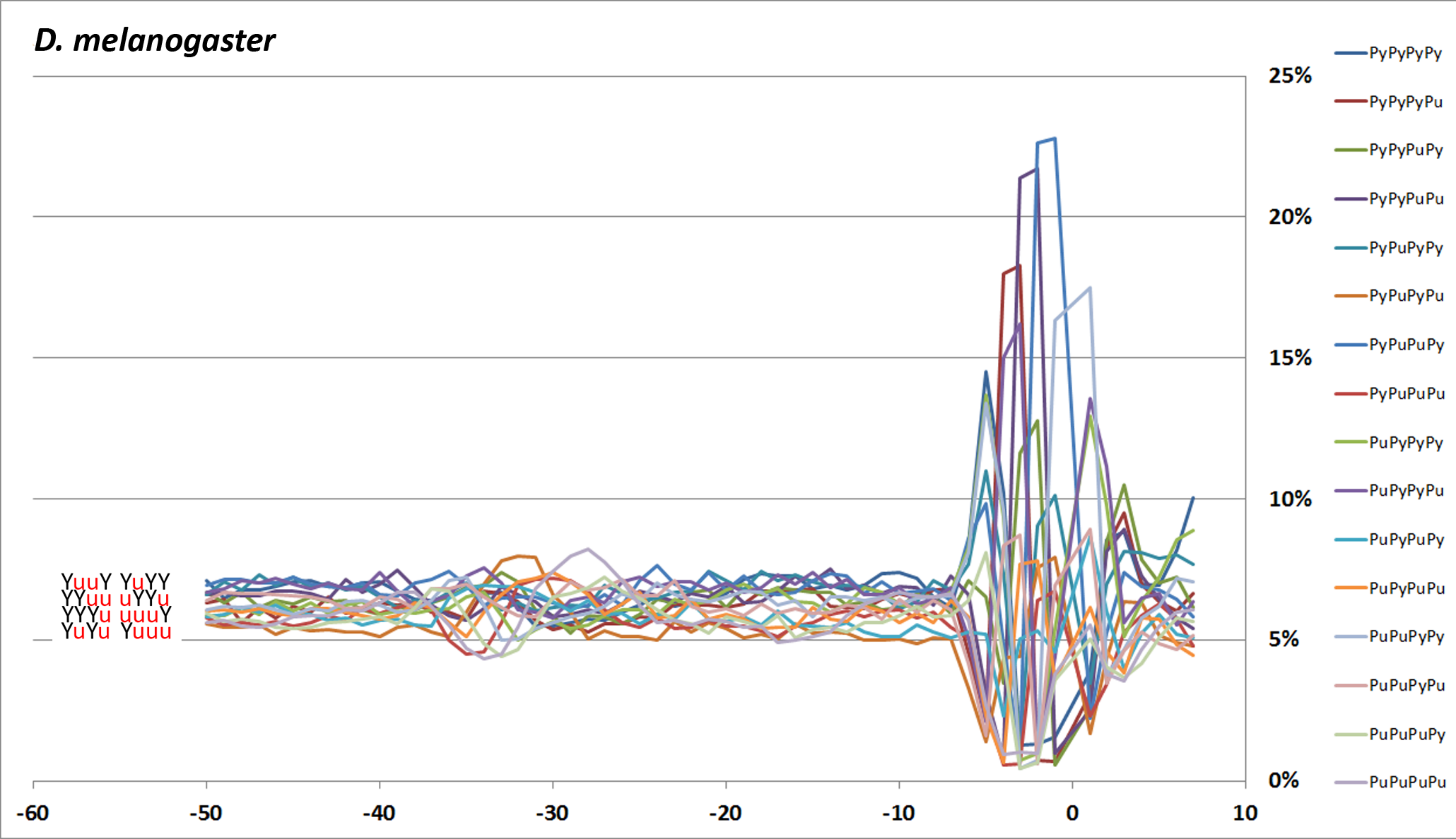
D. melanogaster
25%
20%
15%
10%
5%
0%
-60
-50
-40
-30
-20
-10
0
10
YuuY YuYY
YYuu uYYu
YYYu uuuY
YuYu Yuuu
PyPyPyPy
PyPyPyPu
PyPyPuPy
PyPyPuPu
PyPuPyPy
PyPuPyPu
PyPuPuPy
PyPuPuPu
PuPyPyPy
PuPyPyPu
PuPyPuPy
PuPyPuPu
PuPuPyPy
PuPuPyPu
PuPuPuPy
PuPuPuPu

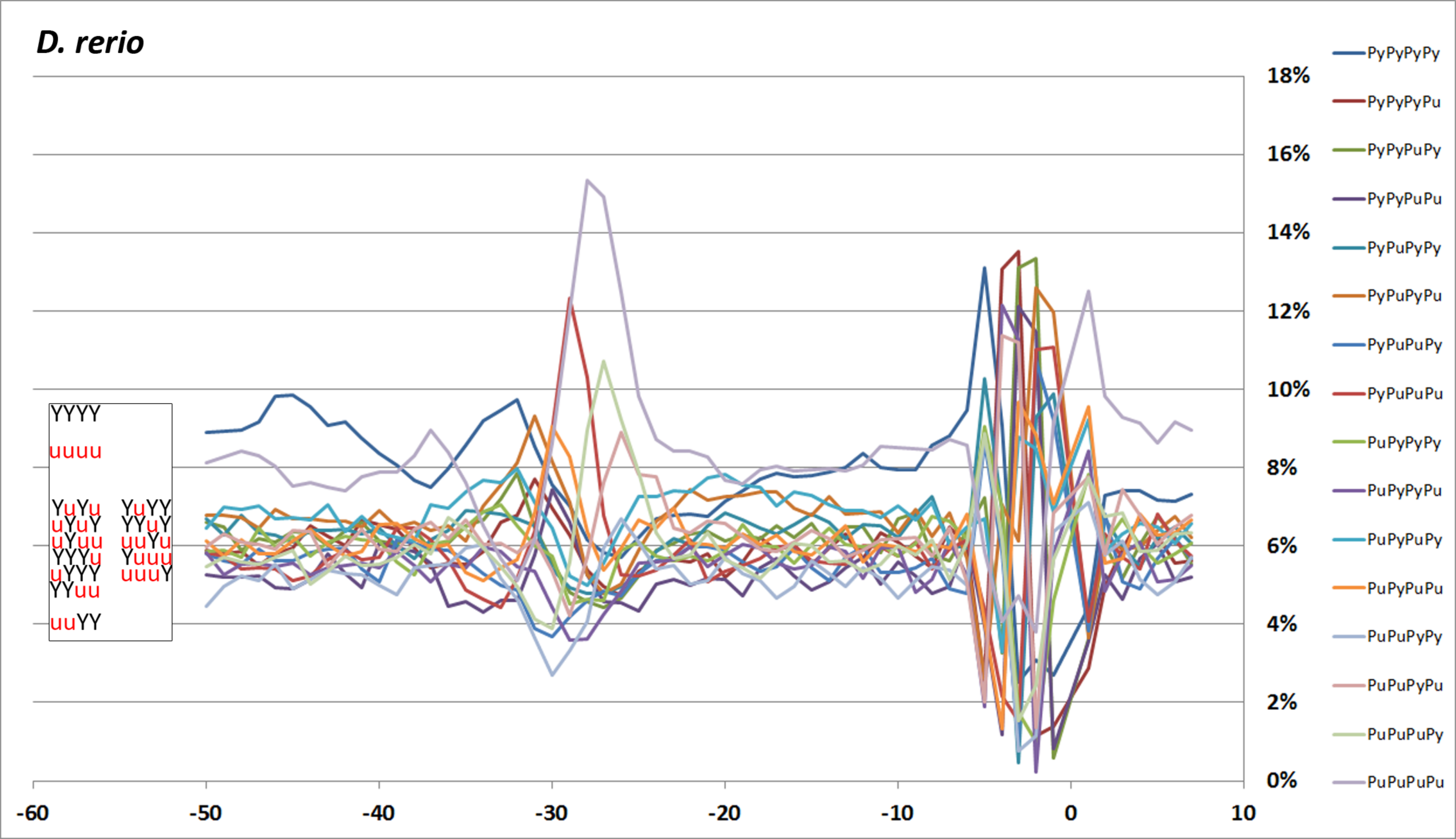

D. rerio
YYYY
uuuu
YuYu
YuYY
uYuY
YYuY
uYuu
uuYu
YYYu
Yuuu
uYYY
uuuY
YYuu
uuYY
18%
16%
14%
12%
10%
8%
6%
4%
2%
0%
-60
-50
-40
-30
-20
-10
0
10
PyPyPyPy
PyPyPyPu
PyPyPuPy
PyPyPuPu
PyPuPyPy
PyPuPyPu
PyPuPuPy
PyPuPuPu
PuPyPyPy
PuPyPyPu
PuPyPuPy
PuPyPuPu
PuPuPyPy
PuPuPyPu
PuPuPuPy
PuPuPuPu

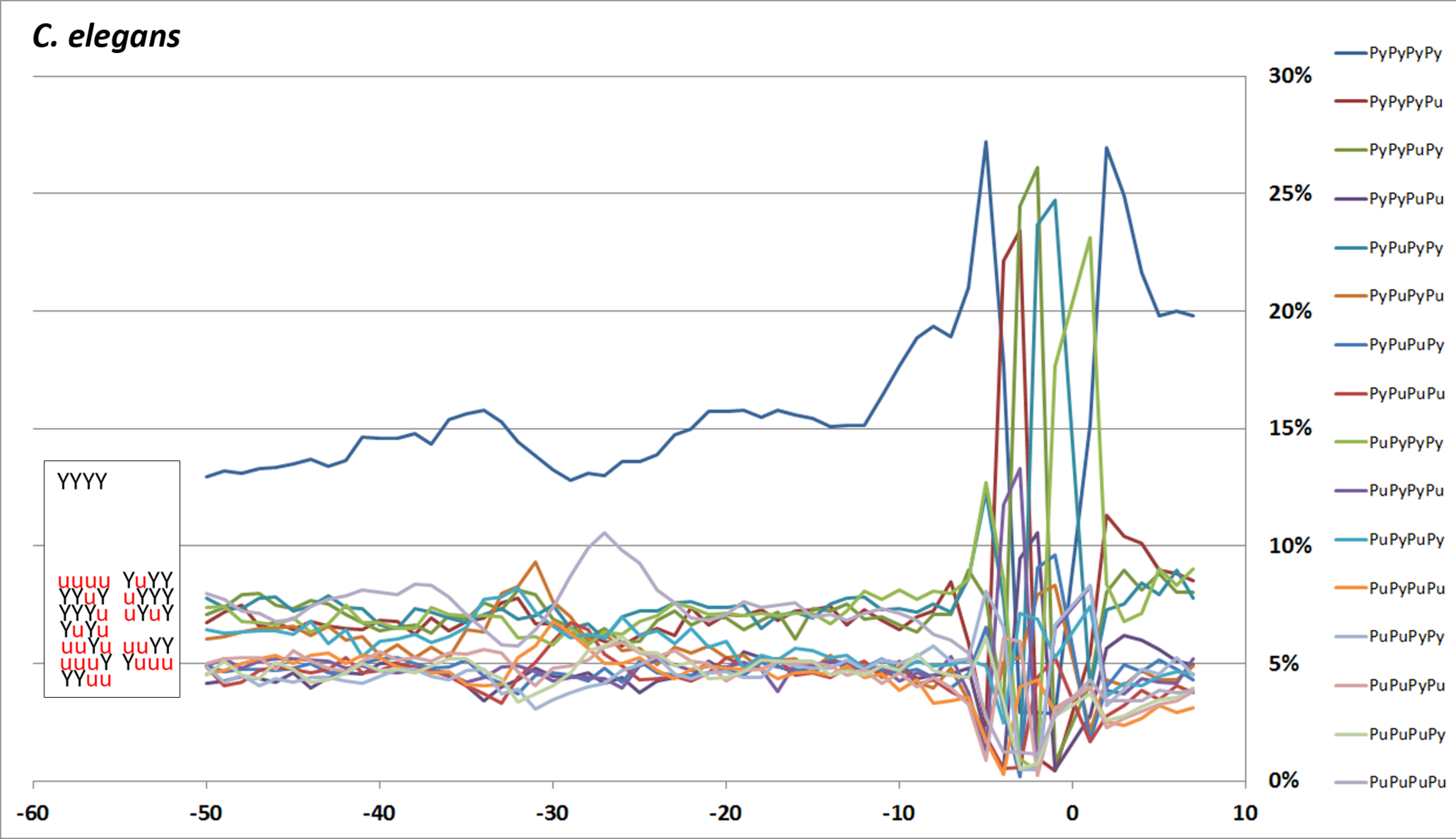
C. elegans
PyPyPyPy
PyPyPyPu
PyPyPuPy
PyPyPuPu
PyPuPyPy
PyPuPyPu
PyPuPuPy
PyPuPuPu
PuPyPyPy
PuPyPyPu
PuPyPuPy
PuPyPuPu
PuPuPyPy
PuPuPyPu
PuPuPuPy
PuPuPuPu
30%
25%
20%
15%
10%
5%
0%
-60
-50
-40
-30
-20
-10
0
10
YYYY
uuuu YuYY
YYuY uYYY
YYYu uYuY
YuYu
uuYu uuYY
uuuY Yuuu
YYuu

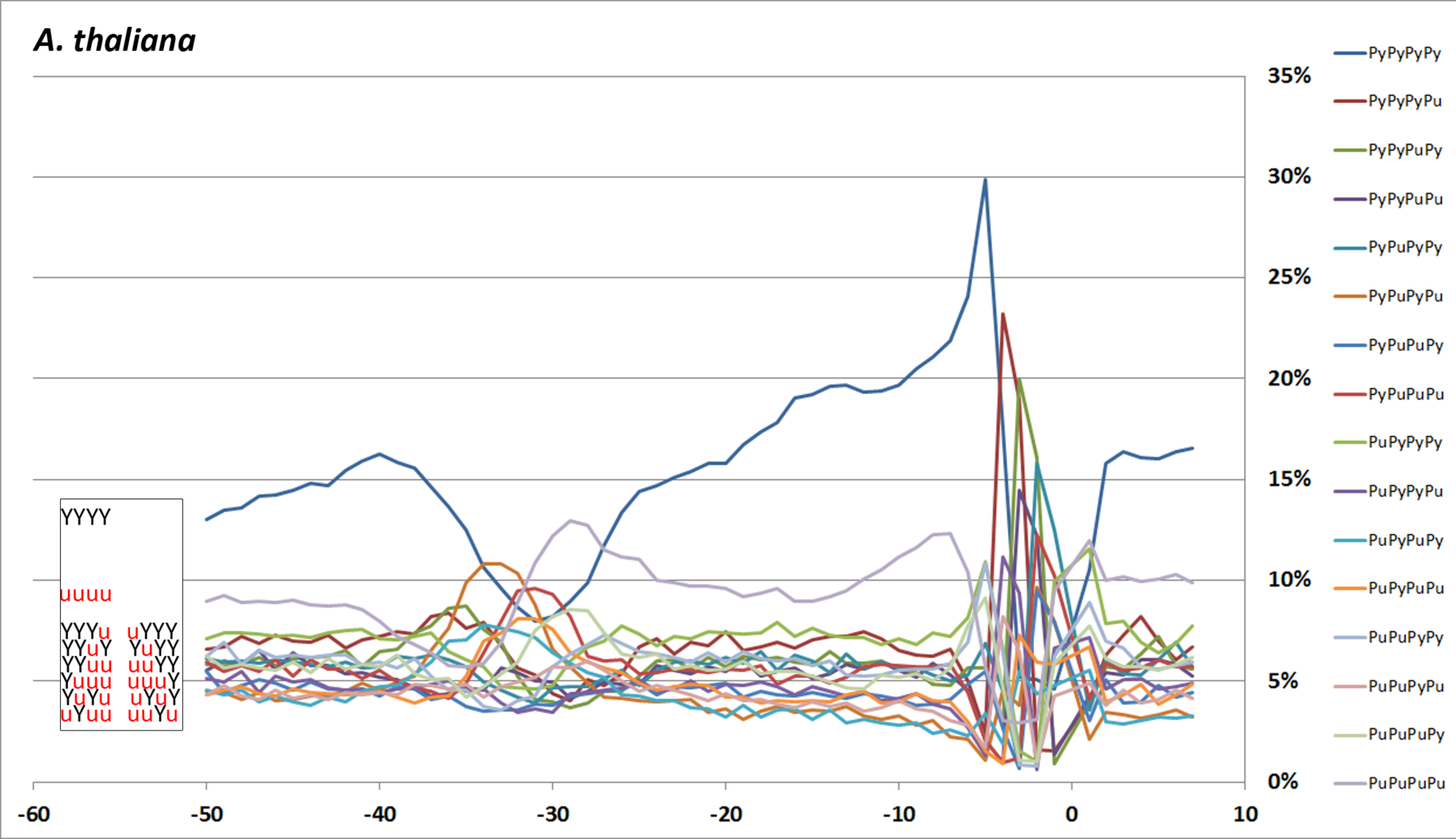

A. thaliana
35%
30%
25%
20%
15%
10%
5%
0%
-60
-50
-40
-30
-20
-10
0
10
PyPyPyPy
PyPyPyPu
PyPyPuPy
PyPyPuPu
PyPuPyPy
PyPuPyPu
PyPuPuPy
PyPuPuPu
PuPyPyPy
PuPyPyPu
PuPyPuPy
PuPyPuPu
PuPuPyPy
PuPuPyPu
PuPuPuPy
PuPuPuPu
YYYY
uuuu
YYYu uYYY
YYuY YuYY
YYuu uuYY
Yuuu uuuY
YuYu uYuY
uYuu uuYu

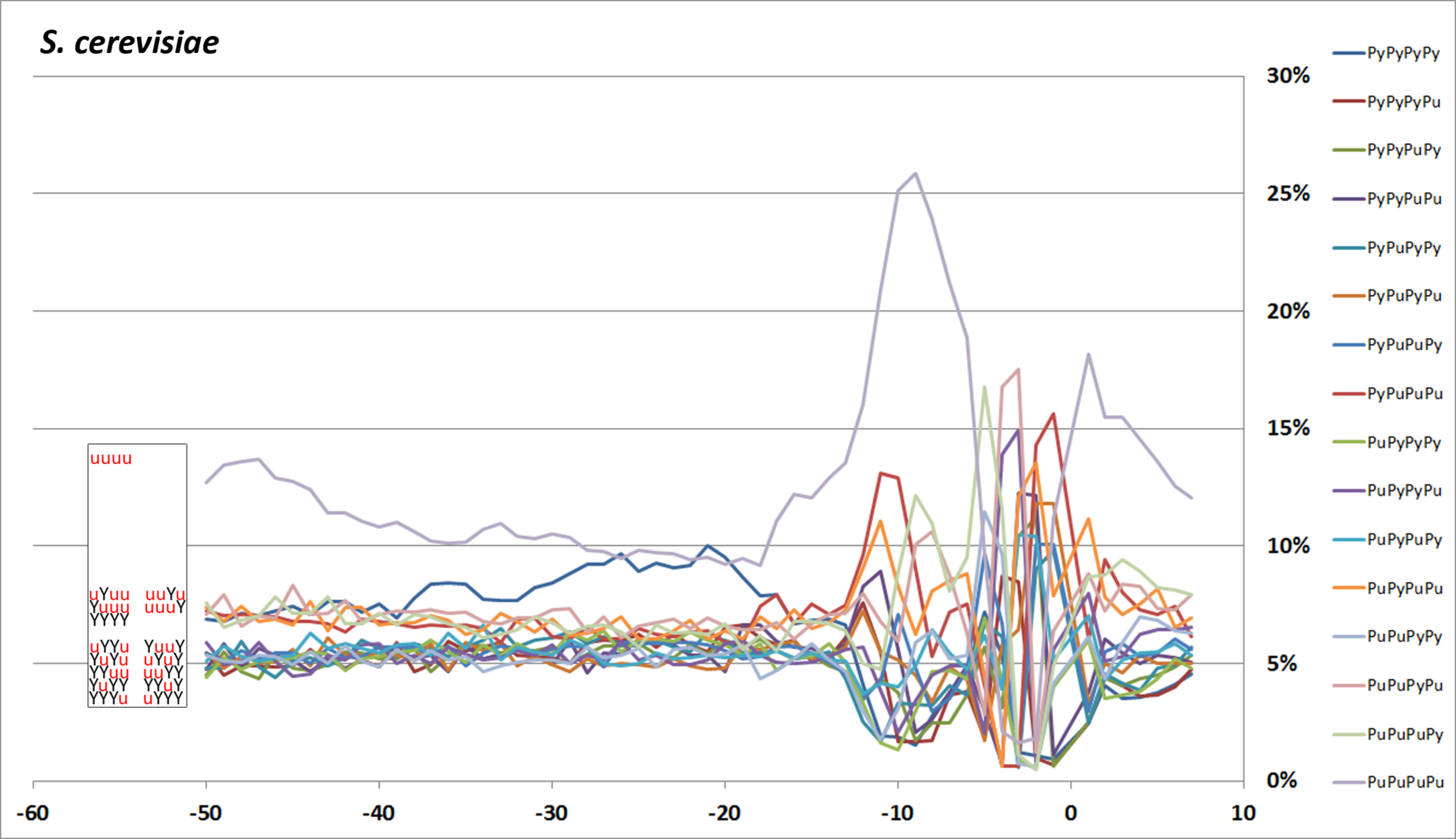

S. cerevisiae
30%
25%
20%
15%
10%
5%
0%
-60
-50
-40
-30
-20
-10
0
10
PyPyPyPy
PyPyPyPu
PyPyPuPy
PyPyPuPu
PyPuPyPy
PyPuPyPu
PyPuPuPy
PyPuPuPu
PuPyPyPy
PuPyPyPu
PuPyPuPy
PuPyPuPu
PuPuPyPy
PuPuPyPu
PuPuPuPy
PuPuPuPu
uuuu
uYuu uuYu
Yuuu uuuY
YYYY
uYYu YuuY
YuYu uYuY
YYuu uuYY
YuYY YYuY
YYYu uYYY

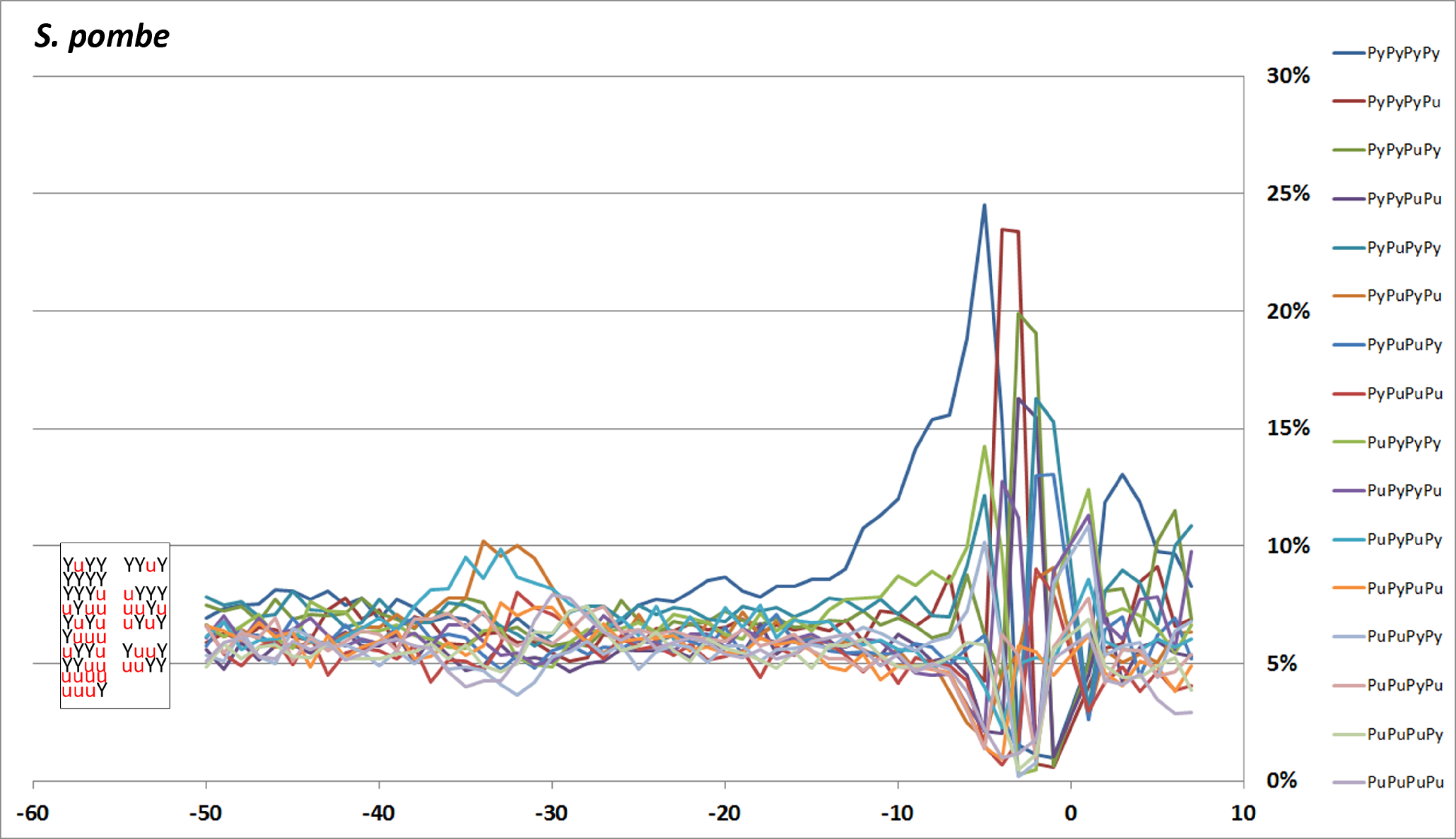
S. pombe
30%
25%
20%
15%
10%
5%
0%
-60
-50
-40
-30
-20
-10
0
10
PyPyPyPy
PyPyPyPu
PyPyPuPy
PyPyPuPu
PyPuPyPy
PyPuPyPu
PyPuPuPy
PyPuPuPu
PuPyPyPy
PuPyPyPu
PuPyPuPy
PuPyPuPu
PuPuPyPy
PuPuPyPu
PuPuPuPy
PuPuPuPu
YuYY YYuY
YYYY
YYYu uYYY
uYuu uuYu
YuYu uYuY
Yuuu
uYYu YuuY
YYuu uuYY
uuuu
uuuY
