# Supplementary material for: Structural features of DNA that determine RNA polymerase II core promoter
Source: BMC Genomics. 2016 Nov 25;17:973. doi: 10.1186/s12864-016-3292-z (PMC5123417; doi:10.1186/s12864-016-3292-z)
Supplement: Additional file 13:Table S1. — is the DataBase for ultrasonic cleavage rates at the dinucleotide level of resolution [23]. (DOCX 17 kb) [file 12864_2016_3292_MOESM13_ESM.docx]

TABLE 1 Sample characteristics of ultrasonic cleavage rates for dinucleotides

________________________________________________

95% confidence limits

|  | N | R | S | SR | Lower limit | Upper limit |
| --- | --- | --- | --- | --- | --- | --- |
| AA | 1636 | 0.919 | 0.129 | 0.003 | 0.913 | 0.926 |
| AC | 1076 | 0.913 | 0.128 | 0.004 | 0.905 | 0.920 |
| AG | 1028 | 0.900 | 0.124 | 0.004 | 0.892 | 0.907 |
| AT | 1374 | 0.904 | 0.119 | 0.003 | 0.898 | 0.910 |
| CA | 1265 | 1.160 | 0.209 | 0.006 | 1.149 | 1.172 |
| CC | 1141 | 1.007 | 0.144 | 0.004 | 0.999 | 1.015 |
| CG | 1230 | 1.444 | 0.334 | 0.010 | 1.426 | 1.463 |
| CT | 1077 | 1.130 | 0.198 | 0.006 | 1.118 | 1.142 |
| GA | 1153 | 0.970 | 0.133 | 0.004 | 0.962 | 0.978 |
| GC | 1317 | 0.954 | 0.146 | 0.004 | 0.947 | 0.962 |
| GG | 1168 | 0.922 | 0.145 | 0.004 | 0.914 | 0.931 |
| GT | 1101 | 0.952 | 0.126 | 0.004 | 0.944 | 0.959 |
| TA | 1065 | 0.973 | 0.120 | 0.004 | 0.966 | 0.980 |
| TC | 1173 | 0.912 | 0.131 | 0.004 | 0.904 | 0.919 |
| TG | 1305 | 0.979 | 0.126 | 0.003 | 0.972 | 0.986 |
| TT | 1672 | 0.932 | 0.127 | 0.003 | 0.938 | 0.938 |

N, sample size; R, mean value; S, standard deviation; SR , standard error

of the mean.
