# Supplementary material for: Structural features of DNA that determine RNA polymerase II core promoter
Source: BMC Genomics. 2016 Nov 25;17:973. doi: 10.1186/s12864-016-3292-z (PMC5123417; doi:10.1186/s12864-016-3292-z)
Supplement: Additional file 14: Table S2. — is the DataBase for ultrasonic cleavage at the tetranucleotide level of resolution [23]. (DOCX 67 kb) [file 12864_2016_3292_MOESM14_ESM.docx]

**Table 2. The sample characteristics of relative intensities of cleavage of central**

**phosphodiester bond in 256 tetranucleotides.**

|  | N | ***T*** | ***S_T_*** | ***S_T_*** | The 95% confidence limits | |
| --- | --- | --- | --- | --- | --- | --- |
|  |  |  |  |  | Lower limit | Upper limit |
| AAAA | 232 | 0.894 | 0.132 | 0.009 | 0.877 | 0.911 |
| AAAC | 151 | 0.883 | 0.139 | 0.011 | 0.860 | 0.905 |
| AAAG | 93 | 0.915 | 0.107 | 0.013 | 0.893 | 0.937 |
| AAAT | 96 | 0.951 | 0.142 | 0.013 | 0.922 | 0.980 |
| CAAA | 114 | 0.921 | 0.169 | 0.012 | 0.890 | 0.952 |
| CAAC | 80 | 0.877 | 0.118 | 0.015 | 0.851 | 0.904 |
| CAAG | 77 | 0.938 | 0.142 | 0.015 | 0.906 | 0.970 |
| CAAT | 77 | 0.935 | 0.126 | 0.015 | 0.907 | 0.964 |
| GAAA | 119 | 0.921 | 0.113 | 0.012 | 0.900 | 0.941 |
| GAAC | 49 | 0.887 | 0.117 | 0.019 | 0.853 | 0.921 |
| GAAG | 88 | 0.936 | 0.113 | 0.014 | 0.912 | 0.960 |
| GAAT | 132 | 0.917 | 0.127 | 0.011 | 0.895 | 0.939 |
| TAAA | 108 | 0.952 | 0.125 | 0.013 | 0.928 | 0.976 |
| TAAC | 73 | 0.916 | 0.097 | 0.015 | 0.894 | 0.939 |
| TAAG | 55 | 0.997 | 0.155 | 0.018 | 0.955 | 1.039 |
| TAAT | 95 | 0.929 | 0.124 | 0.013 | 0.903 | 0.954 |
|  |  |  |  |  |  |  |
| AACA | 96 | 0.938 | 0.112 | 0.011 | 0.915 | 0.960 |
| AACC | 77 | 0.881 | 0.131 | 0.015 | 0.852 | 0.911 |
| AACG | 100 | 0.923 | 0.128 | 0.013 | 0.897 | 0.948 |
| AACT | 78 | 0.919 | 0.127 | 0.014 | 0.890 | 0.947 |
| CACA | 58 | 0.943 | 0.151 | 0.020 | 0.903 | 0.982 |
| CACC | 85 | 0.820 | 0.130 | 0.014 | 0.792 | 0.848 |
| CACG | 46 | 0.871 | 0.174 | 0.026 | 0.819 | 0.922 |
| CACT | 73 | 0.900 | 0.116 | 0.014 | 0.873 | 0.927 |
| GACA | 59 | 0.935 | 0.166 | 0.022 | 0.891 | 0.978 |
| GACC | 34 | 0.921 | 0.117 | 0.020 | 0.880 | 0.962 |
| GACG | 83 | 0.913 | 0.156 | 0.017 | 0.879 | 0.947 |
| GACT | 61 | 0.914 | 0.129 | 0.016 | 0.889 | 0.952 |

| TACA | 38 | 0.986 | 0.094 | 0.015 | 0.937 | 1.013 |
| --- | --- | --- | --- | --- | --- | --- |
| TACC | 79 | 0.887 | 0.113 | 0.013 | 0.862 | 0.913 |
| TACG | 71 | 0.921 | 0.101 | 0.012 | 0.897 | 0.945 |
| TACT | 43 | 0.973 | 0.114 | 0.017 | 0.938 | 1.008 |
|  |  |  |  |  |  |  |
| AAGA | 70 | 0.905 | 0.098 | 0.012 | 0.881 | 0.928 |
| AAGC | 91 | 0.845 | 0.142 | 0.015 | 0.815 | 0.874 |
| AAGG | 72 | 0.924 | 0.133 | 0.016 | 0.893 | 0.955 |
| AAGT | 79 | 0.899 | 0.108 | 0.012 | 0.875 | 0.923 |
| CAGA | 81 | 0.935 | 0.146 | 0.016 | 0.899 | 0.964 |
| CAGC | 100 | 0.846 | 0.136 | 0.014 | 0.819 | 0.873 |
| CAGG | 82 | 0.932 | 0.128 | 0.014 | 0.904 | 0.960 |
| CAGT | 92 | 0.897 | 0.137 | 0.014 | 0.868 | 0.925 |
| GAGA | 50 | 0.880 | 0.118 | 0.017 | 0.846 | 0.914 |
| GAGC | 59 | 0.889 | 0.109 | 0.014 | 0.861 | 0.917 |
| GAGG | 48 | 0.914 | 0.117 | 0.017 | 0.867 | 0.935 |
| GAGT | 50 | 0.874 | 0.107 | 0.015 | 0.844 | 0.905 |
| TAGA | 41 | 0.935 | 0.082 | 0.013 | 0.909 | 0.961 |
| TAGC | 50 | 0.907 | 0.114 | 0.016 | 0.874 | 0.939 |
| TAGG | 40 | 0.886 | 0.145 | 0.023 | 0.840 | 0.933 |
| TAGT | 26 | 0.975 | 0.197 | 0.039 | 0.896 | 1.055 |
|  |  |  |  |  |  |  |
| AATA | 110 | 0.925 | 0.121 | 0.012 | 0.902 | 0.948 |
| AATC | 65 | 0.913 | 0.117 | 0.014 | 0.884 | 0.942 |
| AATG | 98 | 0.913 | 0.127 | 0.013 | 0.887 | 0.938 |
| AATT | 129 | 0.875 | 0.115 | 0.010 | 0.855 | 0.895 |
| CATA | 75 | 0.910 | 0.137 | 0.016 | 0.878 | 0.941 |
| CATC | 67 | 0.801 | 0.189 | 0.023 | 0.755 | 0.847 |
| CATG | 70 | 0.879 | 0.103 | 0.012 | 0.855 | 0.903 |
| CATT | 88 | 0.863 | 0.108 | 0.011 | 0.840 | 0.886 |
| GATA | 91 | 0.907 | 0.083 | 0.009 | 0.890 | 0.924 |
| GATC | 92 | 0.950 | 0.120 | 0.012 | 0.925 | 0.975 |
| GATG | 77 | 0.929 | 0.113 | 0.013 | 0.903 | 0.954 |
| GATT | 76 | 0.913 | 0.107 | 0.012 | 0.888 | 0.937 |
| TATA | 67 | 0.913 | 0.129 | 0.016 | 0.881 | 0.945 |
| TATC | 89 | 0.858 | 0.114 | 0.012 | 0.834 | 0.882 |
| TATG | 69 | 0.946 | 0.106 | 0.013 | 0.920 | 0.971 |
| TATT | 115 | 0.914 | 0.132 | 0.012 | 0.889 | 0.938 |
|  |  |  |  |  |  |  |
| ACAA | 57 | 1.103 | 0.176 | 0.023 | 1.056 | 1.149 |
| ACAC | 53 | 1.108 | 0.177 | 0.024 | 1.059 | 1.156 |
| ACAG | 86 | 1.183 | 0.213 | 0.023 | 1.137 | 1.228 |
| ACAT | 67 | 1.116 | 0.181 | 0.022 | 1.072 | 1.160 |
| CCAA | 73 | 1.025 | 0.178 | 0.021 | 0.984 | 1.067 |

| CCAC | 45 | 0.989 | 0.149 | 0.022 | 0.944 | 1.033 |
| --- | --- | --- | --- | --- | --- | --- |
| CCAG | 96 | 1.190 | 0.213 | 0.022 | 1.147 | 1.233 |
| CCAT | 81 | 1.073 | 0.168 | 0.019 | 1.035 | 1.110 |
| GCAA | 126 | 1.223 | 0.204 | 0.018 | 1.187 | 1.259 |
| GCAC | 71 | 1.174 | 0.215 | 0.026 | 1.123 | 1.225 |
| GCAG | 89 | 1.356 | 0.263 | 0.028 | 1.301 | 1.412 |
| GCAT | 73 | 1.186 | 0.203 | 0.024 | 1.139 | 1.234 |
| TCAA | 93 | 1.127 | 0.171 | 0.018 | 1.092 | 1.162 |
| TCAC | 94 | 1.112 | 0.144 | 0.015 | 1.082 | 1.141 |
| TCAG | 84 | 1.231 | 0.266 | 0.029 | 1.173 | 1.288 |
| TCAT | 80 | 1.129 | 0.200 | 0.022 | 1.085 | 1.174 |
|  |  |  |  |  |  |  |
| ACCA | 78 | 0.975 | 0.112 | 0.013 | 0.949 | 1.000 |
| ACCC | 72 | 0.986 | 0.147 | 0.017 | 0.952 | 1.021 |
| ACCG | 79 | 1.064 | 0.143 | 0.016 | 1.032 | 1.096 |
| ACCT | 45 | 1.012 | 0.101 | 0.015 | 0.981 | 1.042 |
| CCCA | 45 | 0.930 | 0.093 | 0.014 | 0.902 | 0.958 |
| CCCC | 107 | 0.912 | 0.115 | 0.011 | 0.890 | 0.934 |
| CCCG | 103 | 0.992 | 0.147 | 0.014 | 0.964 | 1.021 |
| CCCT | 59 | 0.941 | 0.138 | 0.018 | 0.904 | 0.977 |
| GCCA | 118 | 1.080 | 0.153 | 0.014 | 1.052 | 1.108 |
| GCCC | 50 | 1.085 | 0.223 | 0.032 | 1.022 | 1.148 |
| GCCG | 48 | 1.130 | 0.130 | 0.019 | 1.092 | 1.168 |
| GCCT | 61 | 1.086 | 0.150 | 0.019 | 1.048 | 1.125 |
| TCCA | 53 | 0.999 | 0.127 | 0.018 | 0.964 | 1.035 |
| TCCC | 88 | 0.943 | 0.113 | 0.012 | 0.919 | 0.967 |
| TCCG | 70 | 1.035 | 0.149 | 0.018 | 0.999 | 1.070 |
| TCCT | 70 | 0.966 | 0.130 | 0.016 | 0.935 | 0.997 |
|  |  |  |  |  |  |  |
| ACGA | 86 | 1.537 | 0.331 | 0.036 | 1.466 | 1.608 |
| ACGC | 96 | 1.362 | 0.309 | 0.032 | 1.299 | 1.424 |
| ACGG | 61 | 1.483 | 0.283 | 0.036 | 1.410 | 1.555 |
| ACGT | 53 | 1.381 | 0.369 | 0.051 | 1.279 | 1.482 |
| CCGA | 63 | 1.432 | 0.309 | 0.039 | 1.354 | 1.510 |
| CCGC | 91 | 1.257 | 0.220 | 0.023 | 1.211 | 1.303 |
| CCGG | 91 | 1.417 | 0.311 | 0.033 | 1.352 | 1.482 |
| CCGT | 55 | 1.328 | 0.246 | 0.033 | 1.262 | 1.395 |
| GCGA | 100 | 1.783 | 0.577 | 0.058 | 1.668 | 1.897 |
| GCGC | 87 | 1.462 | 0.358 | 0.038 | 1.385 | 1.538 |
| GCGG | 102 | 1.549 | 0.334 | 0.033 | 1.483 | 1.614 |
| GCGT | 72 | 1.427 | 0.324 | 0.038 | 1.351 | 1.503 |
| TCGA | 47 | 1.543 | 0.371 | 0.054 | 1.434 | 1.652 |
| TCGC | 104 | 1.300 | 0.282 | 0.028 | 1.245 | 1.355 |
| TCGG | 65 | 1.543 | 0.265 | 0.033 | 1.477 | 1.608 |

| TCGT | 58 | 1.263 | 0.227 | 0.030 | 1.203 | 1.323 |
| --- | --- | --- | --- | --- | --- | --- |
|  |  |  |  |  |  |  |
| ACTA | 44 | 1.160 | 0.210 | 0.032 | 1.096 | 1.224 |
| ACTC | 74 | 1.064 | 0.134 | 0.016 | 1.033 | 1.095 |
| ACTG | 58 | 1.157 | 0.243 | 0.032 | 1.093 | 1.221 |
| ACTT | 69 | 1.060 | 0.158 | 0.019 | 1.022 | 1.097 |
| CCTA | 23 | 1.031 | 0.145 | 0.030 | 0.968 | 1.093 |
| CCTC | 73 | 0.980 | 0.128 | 0.015 | 0.950 | 1.010 |
| CCTG | 73 | 1.118 | 0.157 | 0.018 | 1.082 | 1.155 |
| CCTT | 67 | 1.029 | 0.120 | 0.015 | 0.999 | 1.058 |
| GCTA | 40 | 1.227 | 0.166 | 0.026 | 1.173 | 1.280 |
| GCTC | 90 | 1.256 | 0.227 | 0.024 | 1.208 | 1.303 |
| GCTG | 112 | 1.303 | 0.216 | 0.020 | 1.262 | 1.343 |
| GCTT | 88 | 1.210 | 0.225 | 0.024 | 1.162 | 1.257 |
| TCTA | 31 | 1.098 | 0.068 | 0.012 | 1.073 | 1.123 |
| TCTC | 56 | 0.992 | 0.131 | 0.018 | 0.957 | 1.027 |
| TCTG | 101 | 1.149 | 0.195 | 0.019 | 1.111 | 1.188 |
| TCTT | 81 | 1.042 | 0.188 | 0.021 | 1.000 | 1.083 |
|  |  |  |  |  |  |  |
| AGAA | 96 | 0.986 | 0.117 | 0.012 | 0.962 | 1.010 |
| AGAC | 30 | 0.979 | 0.137 | 0.025 | 0.928 | 1.031 |
| AGAG | 48 | 0.956 | 0.144 | 0.021 | 0.914 | 0.998 |
| AGAT | 69 | 1.003 | 0.127 | 0.015 | 0.972 | 1.033 |
| CGAA | 100 | 0.982 | 0.205 | 0.020 | 0.941 | 1.023 |
| CGAC | 84 | 0.914 | 0.150 | 0.016 | 0.881 | 0.946 |
| CGAG | 52 | 0.962 | 0.102 | 0.014 | 0.933 | 0.990 |
| CGAT | 61 | 1.008 | 0.223 | 0.029 | 0.951 | 1.066 |
| GGAA | 102 | 0.968 | 0.123 | 0.012 | 0.943 | 0.992 |
| GGAC | 26 | 0.979 | 0.136 | 0.027 | 0.924 | 1.034 |
| GGAG | 31 | 0.898 | 0.175 | 0.031 | 0.834 | 0.962 |
| GGAT | 98 | 0.998 | 0.155 | 0.016 | 0.967 | 1.029 |
| TGAA | 88 | 0.936 | 0.124 | 0.013 | 0.910 | 0.962 |
| TGAC | 87 | 0.964 | 0.117 | 0.013 | 0.939 | 0.989 |
| TGAG | 77 | 0.997 | 0.106 | 0.012 | 0.973 | 1.021 |
| TGAT | 105 | 0.987 | 0.123 | 0.012 | 0.963 | 1.011 |
|  |  |  |  |  |  |  |
| AGCA | 95 | 0.975 | 0.136 | 0.014 | 0.947 | 1.003 |
| AGCC | 46 | 0.922 | 0.136 | 0.020 | 0.881 | 0.962 |
| AGCG | 73 | 1.029 | 0.136 | 0.016 | 0.997 | 1.060 |
| AGCT | 84 | 0.974 | 0.145 | 0.016 | 0.942 | 1.005 |
| CGCA | 99 | 0.969 | 0.115 | 0.012 | 0.946 | 0.992 |
| CGCC | 106 | 0.886 | 0.173 | 0.017 | 0.853 | 0.919 |
| CGCG | 92 | 0.974 | 0.171 | 0.018 | 0.938 | 1.009 |
| CGCT | 82 | 0.929 | 0.146 | 0.016 | 0.897 | 0.961 |

| GGCA | 75 | 0.913 | 0.128 | 0.015 | 0.884 | 0.943 |
| --- | --- | --- | --- | --- | --- | --- |
| GGCC | 68 | 0.868 | 0.121 | 0.015 | 0.839 | 0.897 |
| GGCG | 104 | 0.967 | 0.169 | 0.017 | 0.934 | 1.000 |
| GGCT | 58 | 0.982 | 0.144 | 0.019 | 0.944 | 1.020 |
| TGCA | 88 | 0.931 | 0.134 | 0.014 | 0.903 | 0.960 |
| TGCC | 55 | 0.958 | 0.143 | 0.019 | 0.919 | 0.996 |
| TGCG | 94 | 1.016 | 0.174 | 0.018 | 0.981 | 1.052 |
| TGCT | 87 | 1.008 | 0.155 | 0.017 | 0.975 | 1.041 |
|  |  |  |  |  |  |  |
| AGGA | 37 | 0.943 | 0.100 | 0.016 | 0.910 | 0.977 |
| AGGC | 70 | 0.905 | 0.150 | 0.018 | 0.869 | 0.941 |
| AGGG | 89 | 0.989 | 0.153 | 0.016 | 0.957 | 1.021 |
| AGGT | 46 | 0.921 | 0.155 | 0.023 | 0.875 | 0.967 |
| CGGA | 89 | 0.962 | 0.112 | 0.012 | 0.938 | 0.985 |
| CGGC | 69 | 0.831 | 0.133 | 0.016 | 0.799 | 0.863 |
| CGGG | 63 | 0.902 | 0.159 | 0.020 | 0.862 | 0.942 |
| CGGT | 100 | 0.925 | 0.148 | 0.015 | 0.895 | 0.954 |
| GGGA | 76 | 0.939 | 0.165 | 0.019 | 0.901 | 0.976 |
| GGGC | 71 | 0.881 | 0.155 | 0.018 | 0.844 | 0.917 |
| GGGG | 84 | 0.958 | 0.160 | 0.018 | 0.923 | 0.993 |
| GGGT | 71 | 0.948 | 0.121 | 0.014 | 0.920 | 0.977 |
| TGGA | 54 | 0.944 | 0.152 | 0.021 | 0.902 | 0.985 |
| TGGC | 93 | 0.859 | 0.142 | 0.015 | 0.830 | 0.888 |
| TGGG | 65 | 0.885 | 0.134 | 0.017 | 0.852 | 0.919 |
| TGGT | 93 | 0.922 | 0.130 | 0.013 | 0.895 | 0.949 |
|  |  |  |  |  |  |  |
| AGTA | 36 | 0.972 | 0.083 | 0.014 | 0.944 | 1.001 |
| AGTC | 38 | 0.909 | 0.170 | 0.028 | 0.853 | 0.965 |
| AGTG | 59 | 0.972 | 0.099 | 0.013 | 0.946 | 0.998 |
| AGTT | 112 | 0.965 | 0.125 | 0.012 | 0.941 | 0.988 |
| CGTA | 60 | 0.987 | 0.081 | 0.010 | 0.966 | 1.008 |
| CGTC | 57 | 0.908 | 0.165 | 0.022 | 0.864 | 0.952 |
| CGTG | 32 | 0.981 | 0.178 | 0.031 | 0.917 | 1.045 |
| CGTT | 91 | 0.940 | 0.138 | 0.014 | 0.911 | 0.968 |
| GGTA | 87 | 0.959 | 0.123 | 0.013 | 0.933 | 0.985 |
| GGTC | 30 | 0.904 | 0.139 | 0.025 | 0.852 | 0.956 |
| GGTG | 88 | 0.966 | 0.134 | 0.014 | 0.938 | 0.995 |
| GGTT | 103 | 0.938 | 0.118 | 0.012 | 0.915 | 0.961 |
| TGTA | 76 | 0.967 | 0.126 | 0.014 | 0.938 | 0.996 |
| TGTC | 62 | 0.921 | 0.127 | 0.016 | 0.888 | 0.953 |
| TGTG | 83 | 0.960 | 0.135 | 0.015 | 0.930 | 0.989 |
| TGTT | 89 | 0.925 | 0.138 | 0.015 | 0.896 | 0.954 |
|  |  |  |  |  |  |  |
| ATAA | 103 | 0.974 | 0.109 | 0.011 | 0.952 | 0.995 |

| ATAC | 94 | 0.963 | 0.148 | 0.015 | 0.933 | 0.994 |
| --- | --- | --- | --- | --- | --- | --- |
| ATAG | 49 | 1.020 | 0.164 | 0.023 | 0.973 | 1.067 |
| ATAT | 98 | 0.945 | 0.136 | 0.014 | 0.918 | 0.972 |
| CTAA | 30 | 1.005 | 0.077 | 0.014 | 0.976 | 1.033 |
| CTAC | 19 | 0.895 | 0.073 | 0.017 | 0.860 | 0.930 |
| CTAG | 24 | 0.998 | 0.065 | 0.013 | 0.970 | 1.025 |
| CTAT | 64 | 0.948 | 0.128 | 0.016 | 0.917 | 0.980 |
| GTAA | 93 | 1.001 | 0.127 | 0.013 | 0.974 | 1.027 |
| GTAC | 43 | 0.986 | 0.130 | 0.020 | 0.946 | 1.026 |
| GTAG | 28 | 1.068 | 0.157 | 0.030 | 1.007 | 1.129 |
| GTAT | 92 | 1.002 | 0.100 | 0.010 | 0.982 | 1.023 |
| TTAA | 103 | 0.925 | 0.111 | 0.011 | 0.904 | 0.947 |
| TTAC | 84 | 0.915 | 0.106 | 0.012 | 0.892 | 0.938 |
| TTAG | 53 | 1.012 | 0.113 | 0.016 | 0.981 | 1.043 |
| TTAT | 86 | 0.960 | 0.104 | 0.011 | 0.937 | 0.982 |
|  |  |  |  |  |  |  |
| ATCA | 104 | 0.967 | 0.165 | 0.016 | 0.935 | 0.999 |
| ATCC | 79 | 0.877 | 0.096 | 0.011 | 0.855 | 0.898 |
| ATCG | 69 | 0.942 | 0.101 | 0.012 | 0.918 | 0.966 |
| ATCT | 64 | 0.941 | 0.110 | 0.014 | 0.913 | 0.968 |
| CTCA | 95 | 0.892 | 0.109 | 0.011 | 0.870 | 0.914 |
| CTCC | 73 | 0.842 | 0.166 | 0.019 | 0.803 | 0.880 |
| CTCG | 51 | 0.937 | 0.107 | 0.015 | 0.906 | 0.967 |
| CTCT | 71 | 0.864 | 0.123 | 0.015 | 0.835 | 0.893 |
| GTCA | 70 | 0.983 | 0.129 | 0.015 | 0.952 | 1.014 |
| GTCC | 36 | 0.932 | 0.080 | 0.013 | 0.905 | 0.959 |
| GTCG | 48 | 0.970 | 0.132 | 0.019 | 0.932 | 1.009 |
| GTCT | 30 | 0.962 | 0.144 | 0.026 | 0.909 | 1.016 |
| TTCA | 81 | 0.917 | 0.115 | 0.013 | 0.891 | 0.942 |
| TTCC | 97 | 0.845 | 0.131 | 0.013 | 0.819 | 0.872 |
| TTCG | 100 | 0.920 | 0.136 | 0.014 | 0.893 | 0.947 |
| TTCT | 99 | 0.905 | 0.136 | 0.014 | 0.878 | 0.932 |
|  |  |  |  |  |  |  |
| ATGA | 85 | 1.017 | 0.105 | 0.011 | 0.994 | 1.039 |
| ATGC | 82 | 0.979 | 0.116 | 0.013 | 0.953 | 1.004 |
| ATGG | 66 | 1.006 | 0.123 | 0.015 | 0.976 | 1.037 |
| ATGT | 80 | 0.951 | 0.137 | 0.015 | 0.920 | 0.981 |
| CTGA | 89 | 1.008 | 0.141 | 0.015 | 0.979 | 1.038 |
| CTGC | 101 | 0.973 | 0.139 | 0.014 | 0.945 | 1.000 |
| CTGG | 82 | 1.008 | 0.109 | 0.012 | 0.984 | 1.032 |
| CTGT | 71 | 0.993 | 0.135 | 0.016 | 0.961 | 1.025 |
| GTGA | 86 | 1.026 | 0.125 | 0.013 | 0.999 | 1.053 |
| GTGC | 73 | 0.929 | 0.139 | 0.016 | 0.897 | 0.962 |
| GTGG | 55 | 1.011 | 0.121 | 0.016 | 0.978 | 1.044 |

| GTGT | 49 | 0.968 | 0.124 | 0.018 | 0.932 | 1.004 |
| --- | --- | --- | --- | --- | --- | --- |
| TTGA | 97 | 0.983 | 0.125 | 0.013 | 0.957 | 1.008 |
| TTGC | 82 | 0.917 | 0.118 | 0.013 | 0.891 | 0.942 |
| TTGG | 102 | 0.960 | 0.132 | 0.013 | 0.934 | 0.986 |
| TTGT | 110 | 0.932 | 0.118 | 0.011 | 0.909 | 0.954 |
|  |  |  |  |  |  |  |
| ATTA | 107 | 0.908 | 0.105 | 0.010 | 0.887 | 0.928 |
| ATTC | 95 | 0.908 | 0.122 | 0.013 | 0.883 | 0.933 |
| ATTG | 95 | 1.004 | 0.116 | 0.012 | 0.980 | 1.027 |
| ATTT | 105 | 0.938 | 0.123 | 0.012 | 0.914 | 0.962 |
| CTTA | 48 | 0.910 | 0.108 | 0.016 | 0.879 | 0.941 |
| CTTC | 90 | 0.895 | 0.098 | 0.010 | 0.874 | 0.915 |
| CTTG | 67 | 0.954 | 0.119 | 0.014 | 0.925 | 0.983 |
| CTTT | 97 | 0.926 | 0.152 | 0.015 | 0.896 | 0.957 |
| GTTA | 81 | 1.041 | 0.145 | 0.016 | 1.009 | 1.073 |
| GTTC | 66 | 0.954 | 0.133 | 0.016 | 0.921 | 0.986 |
| GTTG | 111 | 0.966 | 0.127 | 0.012 | 0.942 | 0.990 |
| GTTT | 138 | 0.945 | 0.125 | 0.011 | 0.924 | 0.966 |
| TTTA | 93 | 0.937 | 0.117 | 0.012 | 0.913 | 0.961 |
| TTTC | 127 | 0.890 | 0.133 | 0.012 | 0.866 | 0.913 |
| TTTG | 117 | 0.932 | 0.119 | 0.011 | 0.910 | 0.954 |
| TTTT | 236 | 0.897 | 0.140 | 0.009 | 0.879 | 0.915 |

Designations: N – the sample size; ***T***- mean value; ***S*** - standard deviation; ***S_T_*** - standard error

of mean.
